# Relating (Un)acceptability to Interpretation. Experimental Investigations on Negation

**DOI:** 10.3389/fpsyg.2017.02370

**Published:** 2018-02-02

**Authors:** Urtzi Etxeberria, Susagna Tubau, Viviane Deprez, Joan Borràs-Comes, M. Teresa Espinal

**Affiliations:** ^1^Centre National de la Recherche Scientifique, IKER (UMR 5478), Bayonne, France; ^2^Departament de Filologia Anglesa i de Germanística, Universitat Autònoma de Barcelona, Barcelona, Spain; ^3^Department of Linguistics, Rutgers University, New Brunswick, NJ, United States; ^4^CNRS, ISC Marc Jeannerod, (UMR 5304), Bron, France; ^5^Department of Spanish Philology, Universitat Autònoma de Barcelona, Barcelona, Spain; ^6^Departament de Filologia Catalana, Universitat Autònoma de Barcelona, Barcelona, Spain

**Keywords:** (un)acceptability, interpretation, negative dependencies, Basque, Castilian Spanish, Basque Country Spanish

## Abstract

Although contemporary linguistic studies routinely use unacceptable sentences to determine the boundary of what falls outside the scope of grammar, investigations far more rarely take into consideration the possible interpretations of such sentences, perhaps because these interpretations are commonly prejudged as irrelevant or unreliable across speakers. In this paper we provide the results of two experiments in which participants had to make parallel acceptability and interpretation judgments of sentences presenting various types of negative dependencies in Basque and in two varieties of Spanish (Castilian Spanish and Basque Country Spanish). Our results show that acceptable sentences are uniformly assigned a single negation reading in the two languages. However, while unacceptable sentences consistently convey single negation in Basque, they are interpreted at chance in both varieties of Spanish. These results confirm that judgment data that distinguish between acceptable and unacceptable negative utterances can inform us not only about an adult’s grammar of his/her particular language but also about interesting cross-linguistic differences. We conclude that the acceptability and interpretation of (un)grammatical negative sentences can serve linguistic theory construction by helping to disentangle basic assumptions about the nature of various negative dependencies.

## Introduction

The notion that grammatical sentences have clear and reliable interpretations across speakers is generally considered a truism that underlies all linguistic inquiry quite independently of the particular theories that given practitioners may favor. On this basis, well-formed or grammatical sentences and their interpretations are considered to be the bricks and mortar with which linguistic theories are built (Chomsky, 1965). Some psycholinguistic studies focus on the comprehension of grammatical sentences that can be difficult to understand due to their linguistic complexity (Gibson and Pearlmutter, 1998), such as garden-path sentences (Frazier and Rayner, 1982). Ferreira et al. (2002) and Ferreira and Patson (2007) argue for the need to undermine the idea that comprehenders always create accurate and detailed representations, and suggest that ‘good enough’ comprehension might be sufficient at the time of understanding grammatical garden-path sentences.

The converse assumption, however —namely that ungrammatical sentences may have unclear and unreliable interpretations across speakers and hence may fail to incite the building of detailed representations— has often been taken as self-evident in linguistics without much discussion, despite being far from consensually agreed upon (Otero, 1972; Shanon, 1973). In fact, several psycholinguistic studies have shown that comprehenders are able to report an initial perception of acceptability and recover an interpretation from ungrammatical sequences (e.g., the amelioration of wh-island violations, Atkinson et al., 2016; the processing facilitation of ungrammatical sequences that contain resumptive pronouns, Beltrama and Xiang, 2016; and the acceptability of semantically implausible sequences that are perceived as plausible sentences, Gibson et al., 2013). Other studies have concentrated on how comprehenders can achieve an interpretation of ungrammatical or ill-formed sentences by covertly correcting the sentence into a well-formed input according to certain principles of repair (Frazier and Clifton, 2011). Also relevant are the series of studies that focus on the perception and comprehension of sequences containing grammatical illusions (such as illusory NPI licensing, Phillips et al., 2011; and comparative illusions, Wellwood et al., 2017). Some of these studies even focus on the ability of adults to learn to comprehend a novel syntactic construction that in strict syntactic terms is ungrammatical (such as the so-called ‘needs’ construction, Kaschak and Glenberg, 2004), a task in which structural and, more specifically, syntactic priming has been claimed to play an important role (Ivanova et al., 2012). Nonetheless, in contrast to the interest aroused by this question in psycholinguistic approaches, in linguistic theory construction the study of the interpretation of ungrammatical and unacceptable sentences is not routinely taken into account.

Here, as linguists, we question this view and ask whether a language speaker’s perception of acceptability (Chomsky, 1965; Schütze, 1996; Sprouse and Almeida, 2013) has any effect on his/her interpretation of sentences, independently of whether the sequences under inspection are grammatical or ungrammatical according to linguistic theory. In itself, the fact that there are ungrammatical sentences that can lead to judgments of acceptability is well-known, since grammaticality and acceptability (the latter being a behavioral measure of the former) have sometimes been seen to diverge (see Lewis and Phillips, 2015). However, whether ungrammatical sentences can lead to reliable interpretations and be informative as to both how sentence meaning is composed and how grammar is involved in this composition is subject to debate in linguistic theorizing. One view has been articulated by Shanon (1973), who, upon analyzing how speakers interpreted sentences that presented a variety of defective agreement patterns, concluded that the interpretation of ungrammatical sentences is not random but rather common to most listeners, and that the process of interpreting ungrammatical sentences appears to be regular and governed by “syntactic, morphophonemic, semantic-pragmatic, and heuristic considerations” (Shanon, 1973, p. 398). In so-called ‘simple cases,’ i.e., in cases in which only one element had to be changed in order to achieve a grammatical sentence, syntactic rules were found to be relevant.

Similarly, in our study we investigate how sentences affected by the addition or removal of only one element —in our experiments the sentential negative marker— are judged as (un)acceptable and interpreted by native speakers of two languages (Basque and Spanish); how stable these judgments and interpretations are within each language; and how they differ across these languages.

In accord with Shanon’s (1973) early experimental work, we argue that the acceptability and interpretation of (un)grammatical negative sentences can inform about the grammar of particular languages and serve linguistic theory construction by helping to disentangle basic assumptions about the nature of various negative dependencies.

A study of the relationship between (un)acceptability and interpretation focusing on the examination of speakers’ judgments about sentences containing negative dependencies appears particularly judicious because this is an area where the syntax, semantics, and pragmatics of language intertwine in a particularly complex and revealing fashion. Indeed, although few concepts could *a priori* seem simpler or more transparent than the logical meaning of negation as an operator that reverses the truth value of a proposition (Horn, 1989), it turns out from a linguistic perspective that negation and negative dependencies can take forms that are really quite diverse and produce interpretations that are surprisingly variable. That is, even though it is clear that all languages feature some designated morpheme or construction that plays a truth-reversing role, i.e., no language fails to feature a sentential negation, not all languages systematically require the overt presence of this marker or construction to build a grammatical sentence that is negative in meaning. That is why it appears to be worth exploring from a linguistic point of view whether the presence or absence of the negative marker is judged as (un)acceptable by native speakers of specific languages, and whether and how its presence or absence affects the interpretation of sentences. To illustrate this phenomenon consider the Standard English sentences in (1).

(1) (a) *Nobody* called.
(b) John did*n’t* call.
(c) *Nobody* did*n’t* call.

In (1a) negation is expressed by means of the negative expression *nobody* and the sentence is interpreted as negative. In (1b) negation is expressed by a negative marker affixed to the verb, and the sentence is also interpreted as conveying single negation (SN), by means of which the truth of the corresponding positive sentence (*John called*) is changed (interpreted as being true when *p* is false, and false when *p* is true). By contrast, in (1c) negation is expressed by a negative expression in subject position in combination with a negative marker, and the interpretation is shifted to what is known as double negation (DN), where two negative expressions cancel each other in standard varieties of English. That is, in these examples the presence or absence of the negative marker is taken to affect not the grammaticality of the sentence but rather the interpretation of the sentence, i.e., it changes the meaning of the sentence from single negation in (1a,b) to double negation in (1c). Grammaticality is here understood in direct relation with a particular interpretation; yet, that link is not always so straightforward in natural languages.

Negation in many languages —be it expressed by means of a sentential negative marker or as part of a possibly negative dependent expression (NDE from now on)^1^— can display a range of morphosyntactic possibilities whose conditions and meaning composition still remain relatively poorly understood despite being widely studied. In this respect, perhaps one of the hardest questions that still arises in the study of negative dependencies is the determination of what exactly can be ‘negative’ across languages and where the meaning of negation actually comes from, be it from the sentential negative marker, directly from a NDE present in the sentence, or from some syntactically determined last resort operator, possibly abstract. This issue relates to the question of what constitutes a well-formed negative sentence, and of when and how sequences showing multiple negative (dependent) expressions can compose into either a SN or a DN interpretation.

In the present study, we comparatively investigate the degree of acceptance of sentences that feature two NDEs (one in subject position and one in object position) in the presence or absence of a sentential negative marker across different languages; we also investigate how these sentences are interpreted, as conveying either a SN or a DN interpretation, as a means to illuminate the cross-linguistic processes that underlie the composition of their negatively related expressions. More specifically, the goal of this paper is to report the results of two online experiments that were designed to test how speakers of two typologically different languages, namely Basque (an isolated language of unknown origin) and Spanish (a Romance language), with two variants for this second language (the variety called Castilian Spanish or Standard Spanish, and the variety of Spanish spoken in the Basque Country, from now on BC-Spanish^2^), judge and interpret negative sentences that combine two NDEs with and without the presence of a sentential negative marker. These two languages were chosen because of the contact situation in which they co-exist, and because they have been described in the literature as featuring NDEs that can variously compose with the presence of an overt negative marker, a phenomenon known in the linguistic literature as Negative Concord (Labov, 1972; van der Wouden and Zwarts, 1993; Giannakidou, 2000; Zeijlstra, 2004, and others).

According to this literature, the presence of an overt negative sentential marker is deemed to be uniformly required for negative dependencies in Basque (Laka, 1990; Etxepare, 2003; de Rijk, 2008), asymmetrically required or precluded in standard Castilian Spanish (Bosque, 1980; Sánchez, 1999), and asymmetrically required but also possible with preverbal NDEs —and hence sometimes optionally available— in BC-Spanish (Franco and Landa, 2006). These three distinct systems are respectively illustrated in (2).

(2) (a) *Inork ez* du *ezer* hautsi. (Basque) anyone.erg not aux anything break ‘Nobody broke anything.’
(b) *Nadie* ha roto *nada*. (Castilian Spanish) nobody has broken anything ‘Nobody broke anything.’
(c) *Nadie (no)* ha roto *nada.* (BC-Spanish) nobody not has broken anything ‘Nobody broke anything.’

Syntactic accounts of these systems usually recognize only two possibilities: the language in question belongs either to the so-called Strict Negative Concord languages, in which the negative marker is always obligatory, or to the so-called Non-Strict Negative Concord languages, where the negative marker is necessarily missing if there is a NDE in preverbal position as in (2b). Systems that manifest optionality as in (2c) are commonly assumed to be a mix of the first two, sometimes conceived as a dialectal variation, other times as an unclear fluctuation effect between the two steady fixed grammar systems.^3^ Such classifications, however, are built on the presupposition that the interpretation of these sentences is that of a single negation. For Spanish, however, the addition of a sentential negation in examples like (2b) has been argued to be ‘ungrammatical’ if the intended meaning is that of single negation, but ‘grammatical’ though poorly acceptable if the meaning is meant to be that of double negation.

However, whether the overt presence of the negative marker is in fact syntactically obligatory, precluded, or optional, or whether its presence or absence in fact affects the interpretation of such sentences rather than their grammatical status is neither fully well-understood nor fully agreed upon. Furthermore, to our knowledge no experimental research has been carried out so far to check what the acceptability judgments and interpretation of (un)grammatical sentences that contain negative dependencies are within a population. Therefore, our main focus here is to systematically couple acceptability judgments with interpretation judgments in order to better clarify how grammar and interpretation interact for the speakers of each of these systems. We focus on the interpretation of Basque sentences without an overt negative marker (taken to be ungrammatical in studies on this language; Euskaltzaindia, 1993), in comparison to Basque sentences with an overt negative marker (taken to be grammatical in the existing literature), and Spanish sentences with an overt negative marker (ungrammatical according to studies on this language; Real Academia Española [RAE], 2009), in comparison to Spanish sentences without an overt negative marker (regarded in the literature as grammatical).

Our initial hypotheses were as follows. (i) For Basque, while we expected sentences without *ez* to be judged unacceptable, we were not sure what their interpretation would be, basically because no previous study has been carried out on the comprehensibility of unacceptable/implausible negative sentences in this language; and we expected that sentences with *ez* would be judged acceptable and interpreted as conveying single negation. (ii) For Castilian Spanish, we expected sentences with *no* to be judged unacceptable and —given the structural and syntactic similarity of negative sentences in Spanish and Catalan, a Romance language on which previous experimental work has been conducted (Déprez et al., 2015; Tubau et al., 2017)— we expected that sentences with *no* in combination with NDEs would facilitate DN readings; at the same time, we expected that sentences without *no* would be judged acceptable and interpreted as expressing single negation. (iii) For BC-Spanish, we expected sentences with *no* to be judged acceptable, due to the presumed optionality illustrated in sentences such as (1c) (taking into account the description by Franco and Landa, 2006), and interpreted as conveying single negation, in parallel to the expectation for Basque; meanwhile, for sentences without *no* we had the same expectations as for Castilian Spanish, i.e., we expected these sentences to be judged acceptable and interpreted as expressing SN.

The paper is organized as follows. In Section “Negative Dependencies in Basque and Spanish” we describe the core characteristics of negation and negative dependencies in Basque and Spanish that are relevant to our study. In Section “Experimental Design” we present the questions that motivated the present research and the experimental design of the two experiments that we ran. In Section “Results” we provide the results of these experiments. A general discussion that synthesizes our core results and holds them up as an illustration of how informative (un)grammatical and (un)acceptable sentences can be for a theory of language closes the paper. In this final section we also discuss some important linguistic conclusions that can be derived from our results for a theory of negation and the phenomenon of Negative Concord.

## Negative Dependencies in Basque and Spanish

In the present paper, we experimentally investigate the acceptability and interpretation of various types of negative dependencies in Basque, Castilian Spanish, and BC-Spanish. However, before we move on to present these experiments, let us briefly describe the grammar of negative sentences in Basque and Spanish, and how grammatical sentences are expected to be interpreted in accordance with the literature on this topic.

Negative concord (NC), a construction widely addressed in the literature of the last four decades (Déprez, 1997; Giannakidou, 2000; Zeijlstra, 2004, and others), features sentences in which two or more (apparently) negative elements co-occur and, crucially, yield a single negation (SN) reading, in apparent violation of strict compositionality, which would demand that two negatives cancel each other out to produce a positive reading. NC, which is commonly attested in a variety of different languages around the world (e.g., Spanish, Italian, Greek, Polish, Russian, West Flemish, Afrikaans, Basque, and many, if not all, the creole languages), is a rather heterogeneous phenomenon in the sense that the properties of the lexical items that participate in it —namely the sentential negative marker and the NDEs— seem to vary in their distribution and composition possibilities from language to language.

Yet, as noted above in connection to the examples in (2), two main kinds of NC have been distinguished on the basis of how the sentential negative marker and NDEs relate in different contexts. NC is defined as Strict (Giannakidou, 1998, 2000) when the sentential negative marker obligatorily co-occurs with both post-verbal and preverbal NDEs. This phenomenon is illustrated in (3) for Basque.

(3) (a) *Inork ez* ditu giltzak galdu. anybody.erg not aux key-D.pl lose ‘Nobody lost the keys.’
(b) Neskak *ez* du *ezer* erosi. girl-D.sg.erg not aux anything buy ‘The girl did not buy anything.’

By contrast, NC is classified as Non-Strict if the sentential negative marker needs to co-occur with post-verbal NDEs, but normally fails to co-occur with preverbal NDEs (unless a DN meaning is intended). Spanish features this kind of NC, (4).

(4) (a) *Nadie* (^∗^*no*) perdió las llaves. nobody not lost the keys ‘Nobody lost the kys.’
(b) La chica *no* compró *nada*. the girl neg bought anything ‘The girl did not buy anything.’

Relevant to our object of study is the fact that for Strict NC languages the absence of the negative marker is associated with ungrammaticality in the linguistic literature, independently of the interpretation of the corresponding utterances by speakers of these languages. For Non-Strict NC languages, in contrast, it is the co-presence of a preverbal NDE with a negative marker that has been claimed to lead to ungrammaticality, but only if a SN reading is intended. As we see, in linguistic theory grammaticality has been understood in direct relation with particular interpretations; yet, that link needs to be clarified against the actual interpretation by speakers of both possible and impossible utterances. Therefore, in the present study, we examine in parallel speakers’ acceptability judgments and their interpretation of negative sentences in two different languages, namely Basque and Spanish.

### Basque

Basque NDEs are morphologically built with wh-words to which the prefix *e-* (>*i-* by dissimilation), is added (Laka, 1990; Euskaltzaindia, 1993; Etxepare, 2003; de Rijk, 2008, etc.).

(5) (a) *i-nor*
prefix *i*-who ‘anybody’
(b) *e-zer*
prefix *e-*what ‘anything’
(c) *i-noiz*
prefix *i-*when ‘any time’
(d) *i-non*
prefix *i-*where ‘anywhere’

In this respect, Basque NDEs appear superficially similar to the English negative quantifiers *no-body, no-thing*, which also combine a negative morpheme within a nominal constituent. Yet, despite the presence of a putative internal negative morpheme, Basque NDEs cannot be used to form a negative sentence by themselves, as the ungrammaticality of (6) with lone NDEs illustrates.

(6) (a) ^∗^Jonek *inor* ikusi zuen. Jon.erg anybody see aux
(b) ^∗^*Inork* goxoki bat jan zuen. anybody.erg candy one eat aux

To be grammatical, Basque NDEs require co-occurrence and composition with a negative marker, (7). This negative marker can in turn license multiple NDEs at once, (8).

(7) (a) *Ez* du *inork* hori erosi. not aux anybody that buy
(b) *Inork ez* du hori erosi. anybody not aux that buy ‘Nobody bought that.’

(8) *Ez* du *inork ezer inon* erosi. not aux anybody.erg anything.abs anywhere buy ‘Nobody bought anything anywhere.’

As they are subject to a stringent licensing requirement by either the sentential negation marker, or some other non-veridical licensor (Zwarts, 1995), it has been suggested that Basque NDEs behave semantically more like non-negative polarity items (Laka, 1990; Etxepare, 2003; de Rijk, 2008), i.e., items with properties akin to those expressions like *anything* in English, than like negative quantifiers, i.e., items akin to *nothing* in English. A problem with this view, however, is that Basque NDEs differ from characteristic polarity items in other respects, as they can and must be licensed by a negation even when they occur in a position that precedes it, as in (7b). Hence, Basque NDEs appear to be semantically non-negative expressions that behave like the NDEs of Strict NC languages, where by definition the negative marker licenses NDEs in all syntactic positions.

Note furthermore that Basque NDEs may appear as pronominal indefinites (*inor* ‘anybody,’ *ezer* ‘anything’), as full NPs (*NP bakar bat ere* lit. NP single one even, where *ere* ‘also, even’ is used and the construction behaves as a minimizer), or as partitive constructions (*NP-etako bakar bat ere* lit. NP D of single one even). The latter two types of NDEs do not bear a negative morpheme, but rather behave similarly to the Basque NPIs that do. All these three types of NDEs were used in our experimental data (see Appendix 1).

### Spanish

Concerning the properties of NDEs in Spanish and their possible co-occurrence with a negative marker, it is commonly claimed that post-verbal NDEs, no matter whether they are subjects or complements, need a co-present negative marker. By contrast, preverbal NDEs can, and preferably do, occur without it.

(9) (a) ^∗^(*No*) sabía *nadie* cuál era la solución. [Sánchez, 1999, p. 2563, ex. (2a)] not knew nobody which was the solution
(b) *Nadie* sabía cuál era la solución. [Sánchez, 1999, p. 2563, ex. (4a)] nobody knew which was the solution ‘Nobody knew the solution.’

Either the sentential negative marker or a subject NDE can license one or more post-verbal NDEs.

(10) (a) *Nadie* sabe *nada*. nobody knows anything
(b) *No* sabe *nadie nada*. not knows anybody anything ‘Nobody knows anything.’

NDEs may appear as argumental pronominal subjects or objects (*nadie* ‘nobody,’ *nada* ‘nothing’), as nominal specifiers (*ningún* X ‘no X’), or as heads of partitive constructions (*ninguno de los* X ‘none of the X’). All three types of negative expressions were used in our experimental data (see Appendix 1).

Spanish sentences that feature a subject and an object NDE, or a sentential negative marker with two or more post-verbal NDEs, are claimed to be most commonly interpreted as expressing SN, the reading associated with NC structures. With preverbal NDEs, however, a co-present sentential negative marker is generally rejected (Bosque, 1980; Sánchez, 1999; Real Academia Española [RAE], 2009), or if accepted at all, often with a particular prosody, it is claimed to yield a DN reading.

(11) *Nadie no* quiere a *nadie*. (^∗^NC) nobody not wants to nobody ‘Nobody likes no one/^∗^anyone.’

Suñer [1995, p. 234, exs. (3a,c)], for example, explicitly claims that the negative marker *no* is incompatible with preverbal negative constituents irrespective of the grammatical function of the NDE in the sentence. She also points out that examples like *Nadie no lo hizo* ‘Nobody didn’t do it’ are possible (though uncommon), but with the two negations canceling each other and producing a meaning equivalent to *Todos lo hicieron* ‘Everybody did it.’

(12) (a) *Nadie* (^∗^*no*) hará eso. nobody not do that ‘Nobody will do that.’
(b) A *ninguno* de ellos (^∗^*no*) llamaría yo. to none of them not call I ‘None of them would I call.’

In BC-Spanish, in contrast, according to Franco and Landa (2006), preverbal NDEs can co-occur with the negative marker and yield a SN reading. Consider (13).

(13) (a) Aquí *nadie no* sabe sobre eso. [Franco and Landa, 2006, p. 2, ex. (4)] here nobody not knows about that ‘Here nobody knows about that.’
(b) Con este alcalde *nada no* tiene sentido. [Franco and Landa, 2006, p. 2, ex. (5)] with this mayor nothing not has sense ‘With this mayor, nothing makes sense.’

The tolerated co-presence of the negative marker with preverbal negative words under a concord reading is likely to stem from a potential Basque influence, as the structural parallelism between (13) and (14) suggests.

(14) (a) *Inork ez* daki hori. anyone.erg not know that.abs ‘Nobody knows that.’
(b) Alkate honekin *ezerk ez* du zentzurik. mayor this.with nothing not aux sense.part ‘Nobody knows that.’

Be this as it may, how acceptable these sentences are for native speakers of this variety and how plausible it is to construct a sensible interpretation in such cases are questions that remain to be investigated.

Before we move on, let us note that the Basque examples in (6) are ungrammatical due to the fact that there is no sentential negative marker in the sentence. This is shown by the star at the left of the example. By contrast, the Spanish sentences in (11) and (12) illustrate a different phenomenon, since these examples are not syntactically ill-formed. According to the literature, these sentences may be uncommon, but are possible if the sentential negation cancels the negation of the preverbal NDE, thus yielding a DN interpretation. Therefore, the negative dependencies in (11) and (12) can be said to be ill-formed only if a SN interpretation is intended. Finally, according to the existing literature, negative dependencies in BC-Spanish are such that the presence of the sentential negative marker is possible with a preverbal NDE, but yields a SN interpretation.

To sum up, negative dependencies are expected to be grammatical in Basque only if they feature the negative marker *ez*. Negative dependencies with preverbal NDEs are expected to be grammatical in Spanish only if the negative marker *no* is absent. Yet, an overt *no* has been described to form grammatical sentences in BC-Spanish. However, there are no claims in the current linguistic literature as to what the predictions regarding acceptability and interpretation are if we reverse the situation, that is, if Basque speakers are faced with sentences without *ez*, and if Spanish speakers (of either Castilian Spanish or BC-Spanish) are faced with sentences with *no.* Acceptability ratings and comprehensibility judgments should provide more qualified information about the knowledge of language held by speakers.

Furthermore, the above noted parallel between Basque and BC-Spanish on the one hand and the contrast between Castilian Spanish and BC-Spanish on the other raise interesting issues about the nature of the concord relation (Strict vs. Non-Strict), regarding how preverbal NDEs can combine and compose with the sentential negative marker and, in particular, why NDEs can sometimes compose with the negative marker and lead to a SN reading but sometimes flip the interpretation to a DN reading. We will discuss these issues in Section “General Discussion.”

## Experimental Design

### Questions

In the present study we sought to explore the following questions related to the acceptability of transitive negative sentences: (i) in the absence of context, how acceptable do speakers judge transitive negative sentences with and without a preverbal negative marker in Basque, Castilian Spanish, and BC-Spanish?; and (ii) are sentences with the negative marker *no* judged more acceptable in BC-Spanish than in Castilian Spanish? If Franco and Landa’s (2006) claim that the negative marker *no* after a preverbal NDE is optional in BC-Spanish is correct, we would expect sentences with *no* to be more acceptable in BC-Spanish than in Castilian Spanish.

Concerning the interpretation of transitive negative sentences in the aforementioned languages, our research questions were the following: (iii) how do speakers interpret transitive negative sentences with and without a preverbal negative marker in Basque and Spanish?; (iv) does the presence vs. absence of the negative marker have any effect on the interpretation of negative sentences in the two languages as should be expected if it has a constant truth reversal meaning?; and (v) are sentences with and without *no* interpreted preferably as conveying single negation in BC-Spanish in comparison to Castilian Spanish? Note that, by focusing on the connection between acceptability and interpretability, we aimed to test the effect of the presence/absence of the overt sentential negative marker not only in a language that is claimed to require it (Basque), but also in two varieties of a language that supposedly do not require it (Castilian Spanish and BC-Spanish). In addition, we also aimed to investigate whether, as claimed by Franco and Landa (2006), the presence of the negative marker *no* after a preverbal NDE in BC-Spanish leads less often to DN than it is expected to do in Castilian Spanish.

Finally, with the aim of looking for correlations between acceptability and interpretation, we also asked question (vi): is there any relation between the mean rate of acceptability and SN/DN interpretation in sentences with and without *ez*/*no* in Basque and Spanish (BC-Spanish and Castilian Spanish)?

These questions motivated the design of two experiments, whose primary goals were (i) to evaluate the extent to which speakers of these languages would accept transitive sentences containing two negative expressions, one preverbal and the other post-verbal with and without the negative marker, and (ii) to find out what interpretations they would attribute to them. In Section “Results” the results of the two experiments, one for Basque (Experiment 1) and one for two varieties of Spanish (Experiment 2), are discussed.

### Method

We designed two experiments, one for Basque and another one for Spanish (Castilian Spanish and BC-Spanish), each consisting of two experimental tasks: an acceptability judgment task and a picture-matching task (Schütze and Sprouse, 2013; Ionin and Zyzik, 2014; Tonhauser and Matthewson, 2015; Juzek, 2016). In the first task, participants were presented with a sentence with no preceding context and had to judge its well-formedness on a five-point Likert Scale after reading the following instruction: “Read the sentence and decide how good it is for a speaker of {Basque, Spanish} on a scale from 1 to 5.” In this gradient scale score 1 corresponded to “least acceptable” and score 5 to “most acceptable.” Immediately after reporting their perception of the acceptability of a given sentence, the participants were directed to the second task, where they were presented with the same sentence and had to choose which of two pictures best represented its meaning. In one picture, none of the characters were performing an action (this picture would correspond to a SN reading); in the other all the characters were performing it (this picture would correspond to a DN reading).

Prior to carrying out the experiment, participants received some training that involved watching a tutorial illustrating a *Likert Scale* and a *Picture-matching* test, but the examples that were used in the training session were unrelated to the items used in the actual experimental task. For each of the experimental stimuli, participants had to perform the two tasks just described. Both tasks required a mouse click (first on the Likert Scale numbers, then on one of the pictures). Once the choice was made, participants could not change their decision, because the second click triggered the appearance of a new test item on the screen.

**FIGURE 1:**
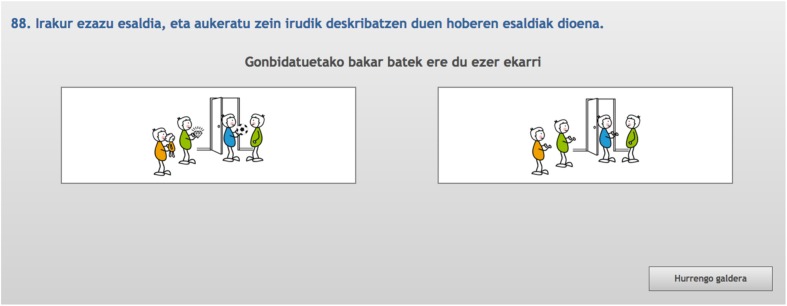
Sample screen of the picture-choice task for the Basque sentence *Gonbidatuetako bakar batek ere du ezer ekarri* lit. guest-D.pl-gen single one.erg even aux anything bring ‘None of the guests brought anything.’ The drawings for the picture-matching task were exactly the same for both experiments. In addition, note that in the particular case of the two varieties of Spanish, participants had to judge exactly the same sentences matched with exactly the same pictures.

A sample screen for the picture-matching task is shown in **Figure 1**, where the picture on the left represents all the individuals performing an action (the DN reading corresponding to the English sentence *None of the guests brought nothing*), while the one on the right represents all of them not performing it (the SN reading corresponding to the English sentence *None of the guests brought anything*).

The experiments were conducted by means of the open access *LingMarket* application that was designed for data gathering by Etxeberria et al. (2013)^4^. All the items were pseudo-randomized via the LingMarket application. The 144 items of the experiment were divided into three blocks: (1) criticals (i.e., test items); (2) controls; and (3) fillers. Each block contained 48 items, which will be described in Section “Materials.” The items in each block were randomized (group-internal randomization of items), but the LingMarket application also incorporates a pseudo-randomization system named “order interleaved sections, random questions in pairs” which allowed us to guarantee, on the one hand, that two critical items would not appear consecutively (to avoid, for example, having a critical sentence with the sentential negative marker being followed by exactly the same sentence without it), and on the other hand, that each participant would view the test items in a different order. This was achieved by programming the system to present one item per block in order, e.g., one item from block 1, one from block 2, one from block 3, one from block 1, one from block 2, one from block 3, and so on.

Whether a picture appeared on the left or the right of the screen during the picture-matching task was also randomized left/right so as to avoid Spatial-Numerical Association of Response Codes (SNARC; Dehaene et al., 1993; Fischer, 2003, and others).

#### Participants

Speakers of Basque took Experiment 1 and speakers of Spanish took Experiment 2. Prior to enrollment, all participants were screened to ensure that they each had a command of the language in which they were going to perform the experiment that was equivalent to at least a European Framework level C. Moreover, all participants were asked to answer a brief sociolinguistic questionnaire at the end of the experiment in which they were asked about their gender, age, place of birth and living area, and their daily percentage of use of their native language. (See the table of demographic information in Appendix 2.)

Experiment 1 was completed by 50 native speakers of Basque,^5^ all students at the University of the Basque Country. For our subsequent statistical analysis, however, we accepted data only from those participants whose percentage of daily use of Basque was 75% or above, which excluded 15 of the initial participants. One additional participant was excluded because less than 75% of his/her responses to control items were correct. Thus, results of Experiment 1 were calculated on the responses of 33 speakers (22 women and 11 men, *M*age = 18.54, *SD* = 1).

Experiment 2 was completed by two groups of Spanish speakers. The first group consisted of 43 speakers of Castilian Spanish, all of them students at the Universidad de Alcalá. However, the responses of 11 of these 43 were excluded from our statistical analysis because they reported a daily use of Spanish that was less than 75%. Of the 32 remaining participants, two more were excluded because fewer than 75% of their responses to control items were correct. Thus, the final statistical analysis was performed on the results of 30 native speakers of Castilian Spanish (26 women and 4 men, *M*age = 21.5, *SD* = 5.9). The second group consisted of 48 speakers of BC-Spanish, all of them Basque-Spanish bilinguals and students at the University of the Basque Country. In this case, data from participants who reported using Spanish less than 50% of the time were excluded, with the result that our final statistical analysis was performed on the responses of only 38 participants (20 women and 18 men, *M*age = 20.39, *SD* = 0.9).

#### Materials

Both Experiment 1 (Basque) and Experiment 2 (Spanish) consisted of 96 transitive stimuli sentences subdivided into eight conditions: four critical conditions (DP-DP, DP-Pro, Pro-DP, and Pro-Pro) and four control conditions (Double Negation, Single Negation Subject, Single Negation Object, and Universal Reading). There were 12 tokens per condition, thus yielding a total of 48 critical items and 48 control items. As the presence or absence of the sentential negative markers *ez* (in Basque) and *no* (in Spanish) was a factor, for each critical condition 6 out of the 12 tokens contained *ez/no* and 6 did not. Thus, we created a factorial design that allowed us to isolate the factors that in the critical conditions could give rise to relative differences in acceptability, namely the presence vs. absence of the negative marker, and differently complex NDEs.

Critical DP-DP stimuli displayed two complex nominal NDEs. Critical Pro-Pro stimuli contained two pronominal NDEs. The other two patterns, namely Pro-DP and DP-Pro, consisted in a combination of a pronominal NDE and a complex DP NDE.

The control Double Negation condition was designed to confirm that our participants could attribute DN readings in the context of a main negative clause and a subordinate negative one, which cancel each other out and yield a positive reading. In Basque what we label Single Negation Object and Single Negation Subject control conditions were designed to confirm that speakers could correctly attribute a SN reading to a sentence containing an object or a subject NDE in the presence of the negative marker *ez*. In the case of Spanish the control Single Negation Object and the control Single Negation Subject were designed to confirm that speakers would attribute a SN reading to a sentence containing a post-verbal NDE c-commanded by the negative marker *no* in the former control, and to a sentence containing a preverbal NDE in the absence of the negative marker *no* in the latter. Finally, it was important to include control Universal Reading items because DN readings can logically correspond to universal quantifier readings, i.e., if there is something that none of the characters in the pictures do not do, then this is something that all of them in fact do.

A total of 48 filler sentences were used as distractors.

The distribution of stimuli is summarized in **Table 1**.

**Table 1:** Syntactic patterns for critical, control conditions, and fillers for both Experiment 1 (Basque) and Experiment 2 (Spanish).

Critical conditions			Control conditions		Fillers
		
#	*ez/no*	Syntactic	#	Syntactic	
		patterns		patterns	
1	√	DP-DP	9	Double Negation	*Transitive sentences with
2	×	DP-DP	10		*no expression of negation
3	√	Pro-Pro	11	Single Negation Object	
4	×	Pro-Pro	12		
5	√	Pro-DP	13	Single Negation Subject	
6	×	Pro-DP	14		
7	√	DP-Pro	15	Universal Reading	
8	×	DP-Pro	16		
	*n* = 6 stimuli	48	*n* = 6 stimuli	48	48


See Appendix 1 for further details about the materials.

#### Procedure

Participants of both Experiments 1 and 2 completed the tasks on an individual computer in a quiet computer room at the University of the Basque Country in Vitoria-Gasteiz (Basque and BC-Spanish groups), or the Universidad de Alcalá (Castilian Spanish group). The test was administered in a single session, and no corrections were allowed. Each experiment lasted between 30 and 40 min.

The participants’ scores given in the 1-to-5 Likert Scale used to measure acceptability were transformed into *z*-scores (Schütze and Sprouse, 2013) to correct for participants’ potentially different treatment of the scale. In our analysis of the data below, we present both Likert scale (LS) scores and *z*-scores but used the latter for purposes of analysis (and accordingly label the variable ‘ZAcceptability’).

All the data obtained were analyzed using a generalized linear mixed model (GLMM) interface within the IBM SPSS Statistics 24 package. The use of Mixed Effects Models allowed us to include random factors for participants and items so that their variation would be appropriately modeled (Baayen et al., 2008; Jaeger, 2008; Barr et al., 2013).

## Results

In this section we present the results from Experiment 1 (Basque) and from Experiment 2 (Castilian Spanish and BC-Spanish), first addressing the research questions related to acceptability (see section “Acceptability”), then the questions related to interpretation (see section “Interpretation”), and finally the issue of possible correlations between interpretation and acceptability (see section “The Relationship between Interpretation and Acceptability”).

### Acceptability

In Experiment 1 (Basque) both control and filler sentences received high acceptability scores (LS mean = 4.453, *SD* = 0.939, mean *z*-score = 0.239, *SD* = 0.668 for controls; and LS mean = 4.722, *SD* = 0.713, mean *z*-score = 0.449, *SD* = 0.530 for fillers). Likewise, in Experiment 2 (Castilian Spanish and BC-Spanish), controls and fillers were given high acceptability ratings by both the Castilian Spanish group (LS mean = 4.465, *SD* = 0.981, mean *z*-score = 0.202, *SD* = 0.755 for controls; and LS mean = 4.760, *SD* = 0.643, mean *z*-score = 0.454, *SD* = 0.544 for fillers) and the BC-Spanish group (LS mean = 4.677, *SD* = 0.798, mean *z*-score = 0.277, *SD* = 0.564 for controls; and LS mean = 4.872, *SD* = 0.484, mean *z*-score = 0.436, *SD* = 0.357 for fillers). (See Table 1 in Appendix 3 for more detailed acceptability results for controls and fillers in the two experiments.)

**FIGURE 2:**
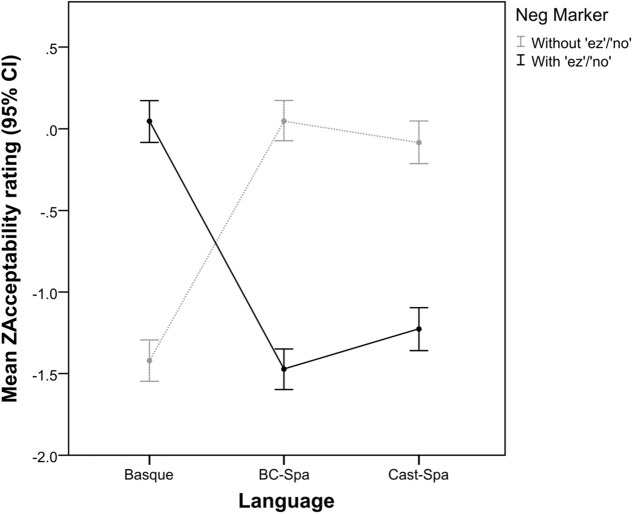
ZAcceptability ratings for Basque, BC-Spanish, and Castilian Spanish by presence vs. absence of the negative marker *ez*/*no.*

Let us now turn to the results for critical items in connection with our research questions (i) and (ii). **Figure 2** shows that, in Basque, sentences with the negative marker *ez* (black solid line) receive high acceptability ratings (mean *z*-score = 0.044, LS mean = 4.199), while sentences without *ez* (light gray dotted line) receive low acceptability ratings (mean *z*-score = -1.421, LS mean = 2.260). For the two varieties of Spanish the situation is the reverse: participants give sentences with the negative marker *no* low acceptability ratings (mean *z*-score = -1.227, LS mean = 2.707 for Castilian Spanish, and mean *z*-score = -1.474, LS mean = 2.514 for BC-Spanish) and sentences without the negative marker *no* high acceptability scores (mean *z-*score = -0.083, LS mean = 4.151 for Castilian Spanish, and *z-*score mean = 0.049, LS mean = 4.396 for BC-Spanish). (See Table 2 in Appendix 3 for more detailed acceptability results for the critical stimuli in the two experiments.)

A Linear Mixed Model was performed on the data, with ZAcceptability set as the dependent variable, and a random intercept defined for both subject and item. The fixed factors were Group (Basque, BC-Spa, Cast-Spa), NegMarker (without *ez*/*no*, with *ez*/*no*), and their interaction.

The results of the fixed effects showed a main effect for NegMarker (*F* = 68.164, *p* < 0.001), but not for Group (*F* = 0.320, *p* = 0.727; see Fixed coefficient results in Appendix 4, Model 1).^6^ The interaction Group × NegMarker was found to be significant (*F* = 377.175, *p* < 0.001), which can be read in the following two ways. On the one hand, Basque speakers preferred sentences with *ez* (*p* < 0.001), whereas the two Spanish groups preferred sentences without *no* (both *p* < 0.001). On the other hand, whereas the acceptability of sentences without *no* was greater for the two Spanish groups (both *p* < 0.001), Basque speakers’ acceptance of sentences with *ez* was greater than the acceptance by the two Spanish groups of sentences with *no* (both *p* < 0.001). Concerning the two Spanish groups, the model did not reveal a significant difference between them for sentences without *no* (*p* = 0.149), but it did for sentences with *no* (*p* = 0.008), which were judged as more acceptable by Castilian Spanish speakers than BC-Spanish speakers.

### Interpretation

In Experiment 1 (Basque), participants interpreted control items correctly 95.2% of the time and filler items 98.4% of the time (see Table 1 in Appendix 3). In Experiment 2 Castilian Spanish speakers interpreted 92.9% of the control items and 98.3% of the filler items correctly, while BC-Spanish speakers interpreted 91.1% of control items and 99.2% of filler items correctly.

**FIGURE 3:**
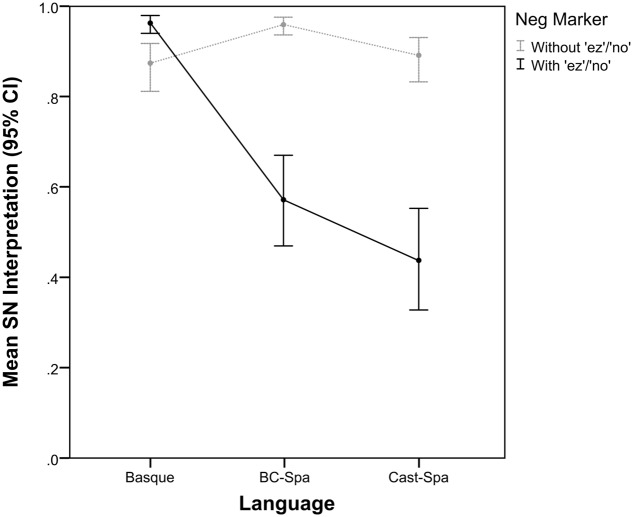
Single negation interpretation for Basque, BC-Spanish, and Castilian Spanish by presence vs. absence of the negative marker *ez*/*no.*

Concerning question (iii), the results for critical stimuli in the three languages are shown in **Figure 3**. As can be seen, in the Basque group, sentences either with or without the negative marker *ez* are most often interpreted as single negation (mean = 0.949 with *ez*, black solid line; mean = 0.840 without *ez*, light gray dotted line), while this is not the case for the two varieties of Spanish. For speakers of either Castilian Spanish or Basque Spanish, sentences without the negative marker *no* are most often interpreted as conveying single negation (mean = 0.849 for Castilian Spanish, and mean = 0.934 for BC-Spanish), while the interpretation of sentences with the negative marker *no* is at chance (mean = 0.446 for Castilian Spanish, and mean = 0.552 for BC-Spanish). (See Table 2 in Appendix 3 for more detailed interpretation results for the critical stimuli in the two experiments.)

To answer questions (iv) and (v), a GLMM was performed on the data, with the response to the image displayed in the picture-matching task (Interpretation) set as the dependent variable (Binomial distribution, Logit link; reference category = DN interpretation). As in the previous model, a random intercept was defined for both subject and item, and Group, NegMarker, and their interaction were set as fixed factors.

The results of the fixed effects showed a main effect for both Group (*F* = 12.457, *p* < 0.001) and NegMarker (*F* = 100.389, *p* < 0.001) (see Fixed Coefficient results in Appendix 4, Model 2). Crucially, their interaction was also found to be significant (*F* = 96.002, *p* < 0.001), which can be interpreted in the following two ways. On the one hand, Basque speakers assigned more SN interpretations to sentences with *ez* (*p* < 0.001), whereas the two Spanish groups assigned more SN interpretations to those sentences without *no* (both *p* < 0.001). On the other hand, several differences appeared between groups when focusing on the two sentence structures. Regarding sentences without *no*, BC-Spanish speakers reported more SN interpretations than Basque speakers did for sentences without *ez* (*p* = 0.018) and Castilian Spanish speakers did for sentences without *no* (*p* = 0.027), with no differences between the latter two (*p* = 0.767). Regarding sentences with *ez*, Basque speakers reported more SN interpretations than the two Spanish groups did for sentences with *no* (both *p* < 0.001), with some tendency for BC-Spanish speakers to provide more SN interpretations than Castilian Spanish speakers, though this difference did not reach significance in our statistical model (*p* = 0.112).

### The Relationship between Interpretation and Acceptability

**FIGURE 4:**
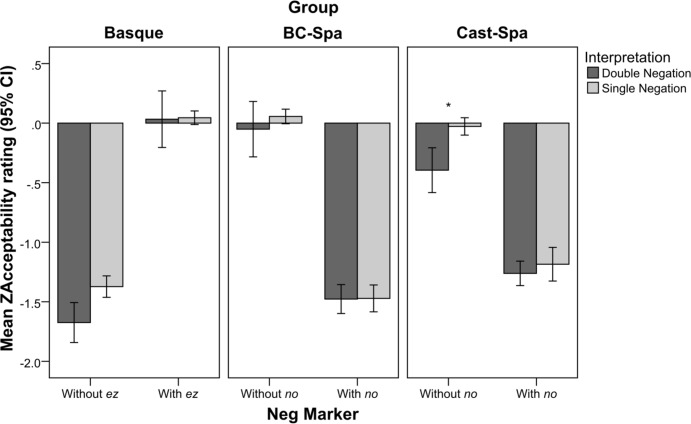
Mean ZAcceptability ratings for Basque, BC-Spanish, and Castilian Spanish by presence vs. absence of the negative marker *ez*/*no* and Interpretation (DN, dark gray; SN, light gray).

**Figure 4** shows the mean ZAcceptability ratings for sentences interpreted as DN (dark gray) and SN (light gray) depending on the presence vs. absence of the negative marker in the languages under study. The star indicates that the difference between sentences interpreted as DN and sentences interpreted as SN in the without *no* condition in Castilian Spanish was found to be statistically significant.

An additional Linear Mixed Model was performed on the data with ZAcceptability (Acceptability ratings converted to z-scores) set as the dependent variable, a random intercept defined both for subject and item, and the following fixed factors: Group (Basque, BC-Spa, Cast-Spa), NegMarker (without *ez*/*no*, with *ez*/*no*), Interpretation (DN, SN), and all their possible interactions.

The effects described in Section “Acceptability” were again found to be significant in the present analysis, namely a main effect for NegMarker (*F* = 27.692, *p* < 0.001), no main effect for Group (*F* = 0.047, *p* = 0.954), and an interaction Group × NegMarker (*F* = 203.604, *p* < 0.001), all of them indicating similar directions of the effects as in the previous results (see Fixed Coefficient results in Appendix 4, Model 3). Two of the effects involving Interpretation were found to be significant: its main effect (*F* = 6.960, *p* = 0.008), and the interaction NegMarker × Interpretation (*F* = 4.610, *p* = 0.032). These effects can be described as follows. The main effect of Interpretation indicates that SN-interpreted sentences received higher acceptability ratings than DN-interpreted ones (β = 0.127, *p* = 0.008); the interaction NegMarker × Interpretation indicates that the preference for higher acceptability ratings for SN interpretations is found when evaluating structures without *ez*/*no* (β = 0.230, *p* = 0.001), but not when *ez*/*no* was present (β = 0.024, *p* = 0.718). The factor Group × Interpretation was not found to be significant (*F* = 1.074, *p* = 0.342), and neither was the triple interaction Group × NegMarker × Interpretation (*F* = 0.958, *p* = 0.384), though its pairwise contrasts are relevant to our research questions to evaluate how the three linguistic groups behaved in relation to the role of acceptability in the interpretation of each sentence structure. In this regard, an interesting finding was that the main effect described for Interpretation was only found for Castilian Spanish when evaluating sentences without *no* (β = 0.403, *p* < 0.001), i.e., sentences without *no* that were interpreted as SN received higher acceptability ratings than those interpreted as DN, but not for any other combination of Group and NegMarker.

## General Discussion

As already claimed in the Introduction, ungrammatical sentences have often been assumed in linguistic theory to lead to unclear and unreliable interpretations across speakers. In this paper, we have questioned this view and have investigated experimentally whether the perception of acceptability (Chomsky, 1965; Otero, 1972) reported by speakers of Basque, Castilian Spanish, and BC-Spanish has any effect on their interpretation of negative sentences, be they grammatical or ungrammatical according to linguistic theory.

### Acceptability and Interpretation of (Un)grammatical Sentences

On the basis of the results obtained in the two experiments, the answer to question (i) (see section “Questions”) is that Basque participants give most items with *ez* high acceptability ratings and most items without *ez* low acceptability ratings. By contrast, Castilian Spanish and BC-Spanish participants give most sentences without *no* high acceptability ratings and sentences with *no* low acceptability ratings. Concerning question (ii), it is not the case that BC-Spanish speakers give sentences with *no* high acceptability ratings (contra the prediction derived from Franco and Landa, 2006). Actually, the mean Acceptability rating for sentences with *no* is lower in BC-Spanish than in Castilian Spanish (LS mean = 2.514, *z*-score mean = -1.474 in BC-Spanish vs. LS mean = 2.707, *z*-score mean = -1.227 in Castilian Spanish). This difference is statistically significant (*p* = 0.008, see Model 1 in Appendix 4), showing that BC-Spanish speakers were more aware of the unacceptability of sentences with *no* than Castilian Spanish speakers.

With regard to question (iii), our results unambiguously show that in Basque SN is the preferred interpretation of sentences either with or without *ez*, while this is only the case for sentences without *no* in BC-Spanish and Castilian Spanish. Moreover, our results show clearly that bilingual Basque/Spanish speakers (i.e., those that ran Experiment 1, and the BC-Spanish group that ran Experiment 2) have a robust grammatical knowledge of the conditions that preferentially license a SN interpretation in the two languages, namely with *ez* in Basque and without *no* in Spanish (see **Figure 3**). For sentences with *no* in the two varieties of Spanish, participants seemed to respond at chance, as shown by a steep decrease in SN readings in favor of DN (in BC-Spanish SN readings: mean = 0.934 for sentences without *no*, mean = 0.552 for sentences with *no*; in Castilian Spanish SN readings: mean = 0.849 for sentences without *no*, mean = 0.446 for sentences with *no*; see **Figure 3** and Table 2 in Appendix 3). Note that the proportion of DN interpretations of critical items was significantly higher than the amount of incorrect responses to control items (correct readings in BC-Spanish controls: mean = 0.911, incorrect readings: mean = 0.089; correct readings in Castilian Spanish controls: mean = 0.929, incorrect readings: mean = 0.071), which suggests that a DN interpretation cannot simply be due to error.

Our results also clearly show that the presence vs. absence of the preverbal negative marker *ez* /*no* plays a crucial role in the interpretation of negative sentences in the three languages under study, which provides a reply to our question (iv). Although the preferred interpretation for critical stimuli was uniformly SN in Basque, sentences with *ez* were more often interpreted as SN than sentences without *ez*, which showed a significant increase in DN readings (*p* < 0.001) (see section “Interpretation of Negative Sentences in Basque and Spanish” for further comment on this issue). In the two varieties of Spanish, by contrast, it was sentences without *no* that received a higher amount of SN interpretation. When comparing the two groups of Spanish speakers [our question (v)], it was found that BC-Spanish participants attributed a higher amount of SN interpretation to sentences without *no* than Castilian Spanish speakers, whereas in sentences with *no*, the two groups behaved alike, merely showing a non-significant tendency to favor SN in BC-Spanish and DN in Castilian Spanish (*p* = 0.112). This confirms that the optionality of the negative marker *no* in BC-Spanish is illusory. BC-Spanish speakers clearly prefer sentences without *no* (again, contra Franco and Landa, 2006), and interpret them similarly to Castilian Spanish speakers.

Furthermore, the behavior of both Castilian Spanish speakers and BC-Spanish speakers concerning sentences without *no* is not parallel. As noted above, both populations judge sentences without *no* as highly acceptable and most often interpret them as SN, but both the acceptability scores (LS mean = 4.396, *z*-score mean = 0.049 in BC-Spanish vs. LS mean = 4.151, *z*-score mean = -0.083 in Castilian Spanish) as well as the SN interpretation rating is higher in BC-Spanish (mean = 0.934) than in Castilian Spanish (mean = 0.849), and there is clearly less dispersion in the responses provided by BC-Spanish speakers, as shown in **Figure 3**. One possible explanation for this may be that, being bilingual speakers of Basque and Spanish, BC-Spanish speakers are aware of both the Basque and the Spanish grammars and, as a result, when they need to interpret sentences without *no*, they manifest a more robust knowledge of the Spanish grammar compared to Castilian Spanish speakers out of a desire to maintain a clear distinction between their two languages. Another possible explanation of the better performance of BC-Spanish speakers is that it reflects normative pressures and is thus not a true grammatical effect.

Finally, concerning question (vi), taking the data of the three language groups together, our results show that there is a relation between acceptability and interpretation. In Basque, BC-Spanish, and Castilian Spanish it is the case that sentences that are interpreted as SN are found to be more acceptable than those that are interpreted as DN. This is especially so for sentences without *no*/*ez*. In Castilian Spanish this tendency reaches statistical significance.

In the particular case of Basque, we conjecture that the decrease in the SN interpretation for sentences without *ez* that have been judged unacceptable may have served the purpose of a non-SN reading rather than a true compositional DN one. In these cases Basque speakers do not seem to be applying a repair strategy that guarantees a SN reading (i.e., the licensing of the NDE), as they seem to be doing for the rest of the data (see section “Interpretation of Negative Sentences in Basque and Spanish”).

The general conclusion is that grammatical sentences are uniformly assigned a SN reading in the three languages; however, ungrammatical sentences convey a clear SN interpretation in Basque, but are interpreted at chance in the two Spanish varieties, with a tendency toward DN in Castilian Spanish and toward SN in BC-Spanish that does not reach statistical significance.

Overall, our experiments also provide interesting results with respect to how negative sentences that are judged (un)acceptable are interpreted in the languages under study. As opposed to theories of categorical grammaticality that assume that only grammatical sentences have representations that can be constructed (Sprouse, 2007), we show that ungrammatical negative sentences can also inform us about the grammar of particular languages. Our data show that sentences judged as unacceptable receive an interpretation that has a variable degree of stability within a given language and furthermore show interesting cross-linguistic differences. This result clearly suggests that the grammar of different languages, in particular the presence vs. absence of sentential negative markers, and the nature of NDEs, influences how both grammatical and ungrammatical sentences are interpreted.

### Interpretation of Negative Sentences in Basque and Spanish

Given the required presence or absence of the negative marker in the diverse cross-linguistic contexts reviewed above, we also aimed at finding out whether negation comes from the negative marker or from the NDE. Is it the nature of the negative marker that cross-linguistically differs from one language to another or is it the particular negative expressions that combine with it? Both hypotheses have been defended in the literature with arguments going back and forth over the decades but not yet culminating in a generally accepted solution. Moreover, why do certain combinations lead to a SN reading in one language and to a DN reading in another? Here, we have explored these questions by testing negative dependencies cross-linguistically with and without the presence of the negative marker. By creating sequences of negative dependencies from which the doubling negation is alternatively present or absent, we tested sentences that in the literature have been said to be either grammatical or ungrammatical in these languages. Beyond this dichotomy, however, the goal was to discover whether interpretations can be stable despite variations in grammaticality and to uncover what the ingredient of interpretation can be in either situation. We expected that the answer to our research questions should allow us to validate at a secondary level some of the predictions made in the literature on Negative Concord (NC), namely (i) that DN readings are available for Strict NC languages (e.g., Basque) only in very restricted morphosyntactic environments (i.e., when two sentential negative markers are distributed in a complex sentence),^7^ and (ii) that DN readings are available in Non-Strict NC languages (e.g., Spanish) when a preverbal NDE co-occurs with the sentential negative marker.

The Basque results for negative sentences with/without *ez* reveal that in some languages DN readings are rarely elicited (even in ungrammatical sentences), providing solid evidence that the nature of so-called NDEs must be non-negative by default. By contrast, the Spanish results for negative sentences with/without *no* show that in other languages DN readings are not altogether implausible.

Why do Basque speakers attribute a SN interpretation to the critical stimuli (see **Figure 3**) in the majority of cases? And why do Spanish speakers behave differently? We hypothesize that in order to answer these questions different types of NDEs must be postulated for the two languages under study. This hypothesis would support some of the claims made in the literature on the expression and interpretation of negation in so-called NC languages (i.e., the distinction between Strict and Non-Strict), but at the same time our results introduce new theoretical issues on the topic, to wit, the nature of NDEs and the licensing of NDEs.

Let us consider a sequence with multiple potentially negative expressions [we exemplify this with a Basque example, repeated from (2a)].

(15) *Inork ez* du *ezer* hautsi. anyone.erg not aux anything break ‘Nobody broke anything.’

A legitimate and central question to ask is what contributes to the expression of single negation in a sentence of this sort, with the two possible logical answers being the sentential negative marker and the NDE itself. Recall that we investigated this question by giving participants sentences that only differed in the presence or absence of the sentential negative marker. The results from Basque support the conclusion that in this language negation comes from the negative marker *ez* and that NDEs are pure negative polarity items (Etxepare, 2003). We know that this is the case because, when two NDEs co-occur in a sentence without *ez*, no DN is obtained beyond what most probably is an error rate, and therefore negation in this language cannot be claimed to come from the NDEs.

Basque speakers are aware of the fact that sentences with two NDEs but no sentential negative marker are not acceptable (they provide low mean acceptability ratings: LS mean = 2.260, *z*-score mean = -1.421; see **Figure 2** and Table 2 in Appendix 3). However, this unacceptability does not prevent them from interpreting the sentences as negative, providing evidence in favor of the idea that the syntactic order of the critical items (sentences without *ez*), where we only eliminated the sentential negative marker but did not change the syntactic order of the rest of the constituents of the sentence, gives enough information for Basque speakers and comprehenders to interpret these sentences as though a sentential negative marker had been overtly realized. This suggests that Basque participants presumably posit an abstract negative operator as soon as they encounter a specific syntactic order and, even though the absence of *ez* will be perceived as priming unacceptability, the sentence will still tend to receive a SN interpretation. To support this conclusion, recall that our experimental items were of the form exemplified in (16), where the two sequences manifest the word order of a negative sentence, differing only in the presence vs. absence of the negative marker *ez*.^8^

(16) (a) *Inork ez* du *ezer* jan. (Grammatical) anyone.erg not AUX anything eat ‘Nobody ate anything.’
(b) ^∗^*Inork* du *ezer* jan. (Ungrammatical) anyone.erg AUX anything eat

For this reason, our results for Basque can be said to make manifest the activation of a repair strategy (Frazier and Clifton, 2011; Phillips et al., 2011; Gibson et al., 2013; Beltrama and Xiang, 2016, and others) and a structural/syntactic priming (Ivanova et al., 2012) that turns a syntactically ungrammatical sentence into a well-formed input where the NDE (a non-negative polarity item in this case) appears to be licensed despite the absence of any overt c-commanding licensor in the syntactic structure (Vasishth et al., 2008). In grammatical terms, in the absence of an overt negative marker *ez*, a null operator NEG, as a last resort option (Zeijlstra, 2004), might be postulated at the syntactic representation of these sentences in order to account for the 31.5% of items that are still considered acceptable but are given a SN reading 83.96% of the time on average (see Table 2 in Appendix 3).

Furthermore, our results show that Basque really is a Strict NC language: since DN is extremely rarely available (considering the results obtained in our experiments) when two NDEs co-occur with the sentential negative marker *ez* within a single sentence, we conclude that the NDEs of this language are non-negative polarity items, but still they are morphosyntactically and semantically different from those found in other Strict NC languages (e.g., Romanian, where a DN reading has been claimed to arise in the presence of two NDEs and a sentential negative marker). Our findings about Basque provide evidence that what is called Strict NC is not uniform across languages. That is, some Strict NC languages allow DN (like Romanian; Falaus and Nicolae, 2016), while others, like Basque, clearly do not. Thus, one must separate the notion of Strict NC from obligatory single negation interpretation.

The results from Castilian Spanish show that negation also comes from the negative marker, since sentences with *no* provoked a correct response in the Single Negation Object control items 95.3% of the times, but in this variety the NDEs may be interpreted as negative as well. Recall that we observed a fluctuation at chance between SN and DN for sentences with *no* (see **Figure 3**). We claim that this result can only be explained if we accept that for some speakers NDEs are negative polarity items (Laka, 1990), but for others are negative quantifiers or negative concord items (Giannakidou, 1998; Herburger, 2001; Espinal and Tubau, 2016), the difference being that the latter type of NDEs can convey a negative meaning. Consequently, when they interact with the sentential negative marker they cancel each other out and entail DN. The claim that NDEs in Castilian Spanish may express negation by themselves is further confirmed when we compare the interpretation of negative sentences without *no* in Castilian Spanish and BC-Spanish (see **Figure 3**). Recall that there is a significant difference between these two variants (*p* = 0.027), which can only be interpreted as indicating that the status of NDEs in these two variants of Spanish is different: they are more polar in BC-Spanish but lean toward a negative quantifier in Castilian Spanish.

The results from BC-Spanish show, again, that negation basically comes from the sentential negative marker. However, these results also indicate that in this variety of Spanish the presence vs. absence of the negative marker is not really optional (contra Franco and Landa, 2006), with our participants showing a clear preference for sentences without *no* (LS mean = 4.396, *z*-score mean = 0.049 for sentences without *no*; LS mean = 2.514, *z*-score mean = -1.474, for sentences with *no*; see **Figure 2** and Table 2 in Appendix 3). On the interpretation side, almost half of the items with *no* were given a DN reading (SN reading, mean = 0.5524; see **Figure 3** and Table 2 in Appendix 3). Since this result is far higher than what we would expect for an error rate, it suggests that the NDEs of this variety may also share the properties of negative quantifiers and negative concord items that we predict for Castilian Spanish.

Hence, our study of the interpretation of (un)grammatical sentences has indirectly provided justification for postulating different types of NDEs and has supported the existence of different types of NC in the two languages. We therefore conclude that differences in the interpretation of ungrammatical sentences point to a difference in the nature of NDEs (negative polarity items in Basque vs. negative quantifiers/negative concord items in both Castilian and BC-Spanish) that was not apparent when only the interpretation of sentences deemed grammatical according to the literature was taken into account. We also conclude that differences in the interpretation of ungrammatical sentences point to a difference in the NC typology. We have confirmed experimentally that Basque is a Strict NC language, whereas the two varieties of Spanish behave as would be expected for Non-Strict NC languages. We have also shown that the grammar of BC-Spanish is closer to Castilian Spanish than to Basque, and that bilingual BC-Spanish/Basque speakers have more robust acceptability and interpretation judgments than monolingual Castilian Spanish speakers.

To sum up, while still upholding the truism that grammatical sentences can lead to reliable interpretations across speakers, our experimental study shows that ungrammatical sentences can also be interpreted reliably, in accordance with the psycholinguistic literature. We conclude that the properties of language-particular grammars do influence the interpretation of ungrammatical sentences and, hence, that such sentences can usefully inform linguistic theory construction.

## Ethics Statement

This study was carried out in accordance with the recommendations of the Regulations of the Universitat Autònoma de Barcelona’s Ethics Committee on Animal and Human Experimentation. The protocol was approved by the Universitat Autònoma de Barcelona’s Ethics Committee on Animal and Human Experimentation of the Univesitat Autònoma de Barcelona (CEEAH-3213). All subjects gave written informed consent prior to their participation.

## Author Contributions

UE ran the experiments (grammaticality judgment task and the picture-matching task) with Basque and Spanish speakers. UE, ST, VD, and MTE designed the experiment, and are responsible for the coding and discussion of the results. ST and JB-C are responsible for the quantitative analysis of the results. MTE is responsible for the whole research.

## Conflict of Interest Statement

The authors declare that the research was conducted in the absence of any commercial or financial relationships that could be construed as a potential conflict of interest.

## Footnotes

1 We use the neutral term ‘negative dependent expression’ because it is important for our empirical investigation to remain agnostic with respect to particular theories.

2 BC-Spanish is the Spanish variety spoken by Basque bilinguals and monolinguals who have lived most of their lives in the Basque Country and whose main Spanish input has always come from persons with similar characteristics.

3 See van der Wouden and Zwarts (1993) and Zeijlstra (2004) for the proposal that the optionality of the negative marker in Catalan correlates with dialectal variation. For a critique of this view see Déprez et al. (2015), Espinal and Tubau (2016), and Tubau et al. (2017).

4 https://isqi.iker.univ-pau.fr/

5 Nowadays all (Central) Basque speakers are bilingual speakers of Basque and Spanish.

6 The IBM SPSS Statistics package gives an output for the fixed effects (which contains an *F*-value and a *p*-value) to estimate the general influence of the fixed factors over all subgroups when a GLMM is run. As our initial interest is in the significance of fixed effects, we first report *F-*values and *p-*values. For those fixed effects that turned out to be significant, we further inspect parameter estimates, which contain β-values, and pairwise contrasts.

7 However, in some Strict NC languages (e.g., Romanian), a DN reading has been claimed to arise in the presence of two NDEs and a sentential negative marker. See Falaus and Nicolae (2016) for the suggestion that DN is also possible in Romanian NDEs used as fragment answers to negative questions.

8 Note that the word order in Basque is SOV, with the auxiliary appearing after the verb in affirmative sentences, and before the verb in negative sentences (see Laka, 1990).
