# Supplementary material for: Relating (Un)acceptability to Interpretation. Experimental Investigations on Negation
Source: Front Psychol. 2018 Feb 2;8:2370. doi: 10.3389/fpsyg.2017.02370 (PMC5801287; doi:10.3389/fpsyg.2017.02370)
Supplement: Supplementary file 1 [file Data_Sheet_1.pdf]

Appendix 1.

A) BASQUE STIMULI – TEST

DP-DP (partitive)

WITH EZ

(1) Ikasleetako bakar batek ere ez du liburuetako
student-D.pl-gen single one-erg even not aux book-D.pl-gen
bakar bat ere irakurtzen.
single one even read
'None of the students has read any of the books.'

(2) Ikasleetako bakar batek ere ez du
student-D.pl-gen single one-erg even not aux
gauzetako bakar bat ere sartu poltsan.
thing-D.pl-gen single one even put bag.in
'None of the students puts any of the things in the bag.'

(3) Lorezainetako bakar batek ere ez du hostoetako bakar bat ere
gardener-D.pl-gen single one-erg even not aux leave-D.pl-gen single one even
hartu.
take
'None of the gardeners grasped any of the leaves.'

(4) Arrantzaleetako bakar batek ere ez du arrainetako bakar bat ere
fisherman-D.pl-gen single one-erg even not aux fish-D.pl-gen single one even
arrantzatu.
catch
'None of the fishermen fished any of the fish.'

(5) Mutikoetako bakar batek ere ez du pilotetako bakar
boy-D.pl-gen single one-erg even not aux ball-D.pl-gen single
bat ere jotzen.
one even hit
'None of the boys hit any of the balls.'

(6) Musikarietako bakar batek ere ez du
musician-D.pl-gen single one-erg even not aux
instrumentuetako bakar bat ere erosi.
instrument-D.pl-gen single one even buy
'None of the musicians bought any of the instruments.'

WITHOUT EZ

(7) Baserritarretako bakar batek ere du behietako bakar
farmer-D.pl-gen single one-erg even aux cow-D.pl-gen single
bat ere elikatu.
one even feed
SN: 'None of the farmers fed any of the cows.'

DN: 'All the farmers fed the cows.'

(8)

Ehiztarietako		bakar	batek	ere	du	txorietako	bakar
hunter-D.pl-gen		single	one-erg	even	aux	bird-D.pl-gen	single
bat	ere	ehizatu.					
one.abs	even	hunt					

SN: 'None of the hunters hunted any of the birds.'
DN: 'All the hunters hunted the birds.'

(9)

Umeetako	bakar	batek	ere	du	kamisetetako	bakar
kid-D.pl-gen	single	one.erg	even	aux	t-shirt-D.pl-gen	single
bat	ere	autsi.				
one.abs	even	break				

SN: 'None of the kids tore any of the t-shirts.'
DN: 'All the kids tore the t-shirts.'

(10)

Umeetako	bakar	batek	ere	du	loreetako	bakar
kid-D.pl-gen	single	one.erg	even	aux	flower-D.pl-gen	single
bat	ere	ukitu.				
one.abs	even	touch				

SN: 'None of the kids touched any of the flowers.'
DN: 'All the kids touched the flowers.'

(11)

Mutikoetako	bakar	batek	ere	du	gauzetako	bakar
boy-D.pl-gen	single	one.erg	even	aux	thing-D.pl-gen	single
bat	ere	bildu.				
one.abs	even	collect				

SN: 'None of the boys collected any of the things.'
DN: 'All the boys collected the things.'

(12)

Mutikoetako	bakar	batek	ere	du	dorreetako	bakar
boy-D.pl-gen	single	one.erg	even	aux	tower-D.pl-gen	single
bat	ere	eraiki.				
one.abs	even	build				

SN: 'None of the boys built any of the towers.'
DN: 'All the boys built the towers.'

Pro-Pro

WITH EZ

(13)

Inork	ez	du	ezer	hautsi.
anyone.erg	not	aux	anything.abs	break

'Nobody broke anything.'

(14)

Inork	ez	du	ezer	ukitu.
anyone.erg	not	aux	anything.abs	touch

'Nobody hid anything.'

(15)

Inork	ez	du	ezer	bildu.

	anyone.erg	not	aux	anything.abs	collect
	‘Nobody collected anything.’				

(16)	Inork	ez	du	inor	ikusi.
	anyone.erg	not	aux	anybody.abs	see
	‘Nobody saw anything.’				

(17)	Inork	ez	du	ezer	botatzen.
	anyone.erg	not	aux	anything.abs	throw.prog
	‘Nobody is throwing anything.’				

(18)	Inork	ez	du	ezer	irakurtzen.
	anyone.erg	not	aux	anything.abs	read.prog
	‘Nobody is reading anything.’				

WITHOUT EZ

(19)	Inork	du	ezer	txukundu.
	anyone.erg	aux	anything.abs	tidy-up.prog
	SN: ‘Nobody is ordering anything.’			
	DN: ‘Everybody is ordering something.’			

(20)	Inork	du	ezer	eraikitzen.
	anyone.erg	aux	anything.abs	build.prog
	SN: ‘Nobody is building anything.’			
	DN: ‘Everybody is building something.’			

(21)	Inork	du	ezer	jaten.
	anyone.erg	aux	anything.abs	eat.prog
	SN: ‘Nobody is eating anything.’			
	DN: ‘Everybody is eating something.’			

(22)	Inork	du	ezer	jotzen.
	anyone.erg	aux	anything.abs	play.prog
	SN: ‘Nobody is playing anything.’			
	DN: ‘Everybody is playing something.’			

(23)	Inork	du	ezer	alokatu.
	anyone.erg	aux	anything.abs	rent.prog
	SN: ‘Nobody rents anything.’			
	DN: ‘Everybody rents something.’			

(24)	Inork	du	ezer	pizten.
	anyone.erg	aux	anything.abs	light.prog
	SN: ‘Nobody lights up anything.’			
	DN: ‘Everybody lights up something.’			

Pro-DP

WITH EZ

(25) Inork ez du abestietako bakar bat ere
anyone.erg not aux song-D.pl-gen single one even
abesten.
sing.prog
'Nobody is singing any of the songs.'

(26) Inork ez du pusketetako bakar bat ere
anyone.erg not aux piece-D.pl-gen single one even
itsasten.
glue.prog
'Nobody is gluing the pieces.'

(27) Inork ez du autoetako bakar bat ere alokatu.
anyone.erg not aux car-D.pl-gen single one even hire
'Nobody has rented any of the cars.'

(28) Inork ez du artelanetako bakar bat ere
anyone.erg not aux artwork-D.pl-gen single one even
ukitzen.
touch.prog
'Nobody is touching any of the artworks.'

(29) Inork ez du kandeletako bakar bat ere
anyone.erg not aux candle-D.pl-gen single one even
pizten.
light.prog
'Nobody is lighting up any of the candles.'

(30) Inork ez du oparietako bakar bat ere ekarri.
anyone.erg not aux present-D.pl-gen single one even bring
'Nobody brought any of the presents.'

WITHOUT EZ

(31) Inork du maletetako bakar bat ere eramaten.
anyone.erg aux suitcase-D.pl-gen single one even carry.prog
SN: 'Nobody is carrying any of the suitcases.'
DN: 'Everybody is carrying the suitcases.'

(32) Inork du kutxetako bakar bat ere mugitzen.
anyone.erg aux box-D.pl-gen single one even move.prog
SN: 'Nobody is moving any of the boxes.'
DN: 'Everybody is moving the boxes.'

(33) Inork du liburuetako bakar bat ere jaso.
anyone.erg aux book-D.pl-gen single one even tidy-up.prog
SN: 'Nobody collects any of the books.'
DN: 'Everybody collects the books.'

(34) Inork du dorreetako bakar bat ere eraikitzen.
anyone.erg aux tower-D.pl-gen single one even build.prog
SN: 'Nobody builds any of the towers.'
DN: 'Everybody builds the towers.'

(35) Inork du liburuetako bakar bat ere irakurtzen.
anyone.erg aux book-D.pl-gen single one even read.prog
SN: 'Nobody is reading any of the books.'
DN: 'Everybody is reading the books.'

(36) Inork du lorontzietako bakar bat ere botatzen.
anyone.erg aux vase-D.pl-gen single one even throw.prog
SN: 'Nobody throws any of the vases.'
DN: 'Everybody throws the vases.'

DP-Pro

WITH EZ

(37) Jokalarietako bakar batek ere ez du ezer
player-D.pl-gen single one.erg even not aux anything
botatzen.
throw.prog
'None of the players is throwing anything.'

(38) Gonbidatuetako bakar batek ere ez du ezer
guest-D.pl-gen single one.erg even not aux anything
jaten.
eat.prog
'None of the guests is eating anything.'

(39) Umeetako bakar batek ere ez du ezer
kid-D.pl-gen single one.erg even not aux anything
edaten.
drink.prog
'None of the children is drinking anything.'

(40) Ikasleetako bakar batek ere ez du ezer
guest-D.pl-gen single one.erg even not aux anything
idazten.
write.prog
'None of the students is writing anything.'

(41) Bisitarietako bakar batek ere ez du ezer
visitor-D.pl-gen single one.erg even not aux anything
lapurtzen.
steal.prog
'None of the visitors is stealing anything.'

(42) Jostunetako bakar batek ere ez du ezer

tailor-D.pl-gen single one.erg even not aux anything
korapilatzen.
tangle.prog
'None of the tailors is tangling anything.'

WITHOUT EZ

(43) Umeetako bakar batek ere du ezer altxatzen.
kid-D.pl-gen single one.erg even aux anything lift.prog
SN: 'None of the children is lifting anything.'
DN: 'All the children are lifting something.'

(44) Gonbidatuetako bakar batek ere du ezer ekarri.
guest-D.pl-gen single one.erg even aux anything bring
SN: 'None of the guests brought anything.'
DN: 'All the guests brought something.'

(45) Umeetako bakar batek ere du ezer eraikitzen.
kid-D.pl-gen single one.erg even aux anything build.prog
SN: 'None of the children is building anything.'
DN: 'All the children are building something.'

(46) Ikasleetako bakar batek ere du ezer
guest-D.pl-gen single one.erg even aux anything
eramaten.
carry.prog
SN: 'None of the students is carrying anything.'
DN: 'All the students are carrying something.'

(47) Ikasleetako bakar batek ere du ezer
guest-D.pl-gen single one.erg even aux anything
pizten.
light.prog
SN: 'None of the students lights up anything.'
DN: 'All the students light up something.'

(48) Gonbidatuetako bakar batek ere du ezer
guest-D.pl-gen single one.erg even aux anything
jasotzen.
tidy-up.prog
SN: 'None of the guests tidies up anything.'
DN: 'All the guests tidy up something.'

CONTROL DOUBLE NEGATION

(49) Ez da egia langileek ez dutenik ezer
not is truth worker-D.pl-erg not comp anything
lurperatu.
bury
'It is not true that the workers have not buried anything.'

(50) Ez da egia arrantzaleek ez dutenik arrain bakar
not is truth fisherman-D.pl-erg not comp fish single
bat ere arrantzatu.
one even catch
‘It is not true that the fishermen have not fished anything.’

(51) Ez da egia ikasle bakar batek ere ez duenik
not is truth studentsingle one.erg even not comp
bozkatu.
vote
‘It is not true that no student has voted.’

(52) Ez da egia gonbidatu bakar batek ere ez duenik
not is truth guest single one.erg even not comp
jan.
eat
‘It is not true that no guest has eaten.’

(53) Ez da egia ume bakar batek ere ez duenik
not is truth kid single one.erg even not comp
ezer altxatzen.
anything say.prog
‘It is not true that no child lifted anything.’

(54) Ez da egia ume bakar batek ere ez duenik pieza
not is truth kid single one.erg even not comp piece
bakar bat elkartu.
single one join
‘It is not true that no child assembled any piece.’

(55) Ez da egia inork ez duenik leihoa ireki.
not is truth anybody.erg not comp window open
‘It is not true that nobody opened the window.’

(56) Ez da egia inork ez duenik lora bat erosi.
not is truth anybody.erg not comp flower one buy
‘It is not true that nobody bought a flower.’

(57) Ez da egia inork ez duenik liburu bat jaso.
not is truth anybody.erg not comp book one lift
‘It is not true that nobody collects a book.’

(58) Ez da egia inork ez duenik kandela bat piztu.
not is truth anybody.erg not comp candle one light
‘It is not true that nobody lights up a candle.’

(59) Ez da egia inork ez duenik ardoa edan.
not is truth anybody.erg not comp wine drink
‘It is not true that nobody drinks wine.’

(60) Ez da egia inork ez duenik gozo bat jan.
not is truth anybody.erg not comp sweet one eat
'It is not true that nobody eats a sweet.'

CONTROL SINGLE NEGATION SUBJECT

(61) Inork ez dio lehenengo mutikoari jarraitzen.
anybody not aux first boy.D.sg.dat follow.prog
'Nobody is following the child.'

(62) Bisitari bakar batek ere ez du harribitxi bat
visitor single one.erg even not aux gem one
begiratzen.
see.prog
'No visitor is looking at any jewel.'

(63) Inork ez ditu giltzak galdu.
anybody.erg not aux key-D.pl lose
'Nobody lost the keys.'

(64) Langile bakar batek ere ez du zulo bat
worker single one.erg even not aux hole one
egiten.
do.prog
'No worker is making a hole.'

(65) Inork ez ditu eskolako gauzak poltsan sartzen.
anybody.erg not aux school.gen things bag.in introduce.prog
'Nobody puts the school things in the bag.'

(66) Ume bakar batek ere ez du orri bat goraizekin
kid single one.erg even not aux paper one scissors.with
mozten.
cut.prog
'No child is cutting a paper with the scissors.'

(67) Inork ez ditu maletak ekarri.
anybody.erg not aux suitcases bring
'Nobody brought the suitcases.'

(68) Ikasle bakar batek ere ez dauka orri
student single one.erg even not aux.have page
margotu bat.
painted one
'No student has a painted sheet.'

(69) Inork ez ditu gozoak jaten.
anybody.erg not aux sweets eat.prog

‘Nobody eats the sweets.’

(70) Ikasle bakar batek ere ez du kamixeta
student single one.erg even not aux t-shirt
hausten.
break.prog
‘No student tears his t-shirt.’

(71) Inork ez du lorontzi bat botatzen.
anybody.erg not aux vase one throw.prog
‘Nobody throws a vase.’

(72) Ume bakar batek ere ez du ura edaten.
kid single one.erg even not aux water drink.prog
‘No kid drinks wine.’

CONTROL SINGLE NEGATION OBJECT

(73) Mutikoak ez du inor ikusi.
boy-D.sg.erg not aux anybody see
‘The boy didn’t see anybody.’

(74) Bisitariak ez du harribitxi bakar bat ere
visitor-D.sg.erg not aux gem single one even
begiratzen.
see.prog
‘The visitor doesn’t see any gem.’

(75) Neskak ez du ezer erosi.
girl-D.sg.erg not aux anything buy
‘The girl didn’t buy anything.’

(76) Langileak ez du zulo bakar bat ere egiten.
worker-D.sg.erg not aux hole single one even do.prog
‘The worker doesn’t dig any hole.’

(77) Ikasleek ez du poltsan ezer sartzen.
student-D.pl.erg not aux bag.in anything introduce.prog
‘The students don’t put anything in the bag.’

(78) Umeak ez du goraizekin ezer mozten.
kid-D.sg.erg not aux scissors.with anything cut.prog
‘The child doesn’t cut anything with the scissors.’

(79) Gizonak ez du maleta bakar bat ere ekartzen.
man-D.sg.erg not aux suitcase single one even bring.prog
‘The man doesn’t bring any suitcase.’

(80) Ikasleak ez du ezer margotzen.
student-D.sg.erg not aux anything paint.prog

‘The student doesn’t paint anything’

(81) Mutikoek ez dute ezer jaten.
boy-D.pl.erg not aux anything eat.prog
‘The boy doesn’t eat anything.’

(82) Bisitariak ez du koadro bakar bat ere
visitor-D.sg.erg not aux painting single one even
ukitzen.
touch.prog
‘The visitor doesn’t touch any painting.’

(83) Neskak ez du ezer edaten.
girl-D.sg.erg not aux anything drink.prog
‘The girl didn’t drink anything.’

(84) Ikasleak ez du kandela bakar bat ere
student-D.sg.erg not aux candle single one even
pizten.
light.prog
‘The student doesn’t light up any candle.’

CONTROL UNIVERSAL READING

(85) Ikasle guztiek zerbait idazten dute.
student all-D.pl.erg something write.prog aux
‘All students are writing something.’

(86) Jonek objektu guztiak ukitzen ditu.
Jon.erg object all-D.pl touch.prog aux
‘Jon is touching the objects.’

(87) Denek zerbait ekarri dute.
all-D.pl.erg something bring aux
‘Everybody brought something.’

(88) Anek gauza guztiak mugitzen ditu.
Ane.erg thing all-D.pl move.prog aux
‘Ane is moving all the things.’

(89) Umeek orri guztiak margotzen dituzte.
kid-D.pl.erg page all-D.pl paint.prog aux
‘The children are painting all the sheets.’

(90) Denek bozka bat eman dute.
all-D.pl.erg vote one give aux
‘Everybody gave his/her vote.’

(91) Langileek zulo guztiak egin dituzte.
worker-D.pl.erg hole all-D.pl do aux

‘The workers dug all the holes.’

(92) Langile bakoitzak bere lana egiten du.
worker each-D.sg.erg his/her job do.prog aux
‘Each worker did his job.’

(93) Denek zerbait edan dute.
all-D.pl.erg something drink aux
‘Everybody drank something.’

(94) Anek koadro guztiak margotu ditu.
Ane.erg painting all-D.pl paint aux
‘Ane painted all the paintings.’

(95) Ikasle guztiek zerbait pizten dute.
student all-D.pl.erg something light aux
‘All the students light up something.’

(96) Jonek gauza guztiak poltsan sartzen
Jone.erg painting all-D.pl bag.in introduce.prog
ditu.
aux
‘Jone puts all the things in the bag.’

FILLERS

(97) Botanikariek hostoak ikertzen dituzte.
botanist-D.pl.erg leaves investigate.prog aux
‘Botanists are investigating plants.’

(98) Umeek telesaio bat ikusten dute.
kid-D.pl.erg tv show one watch.prog aux
‘The children are watching a series.’

(99) Ikasleek zerbait irakurtzen dute.
estudiante-D.pl.erg something read.prog aux
‘The students are reading something.’

(100) Ikasle bakoitzak jarduera desberdin bat egiten
Student each-D.sg.erg activity different one do.prog
du.
aux
‘Each student is doing a different activity.’

(101) Bozka-emaileak bozkatzen ari dira.
voter-D.pl.erg vote.prog prog aux
‘Voters are voting.’

(102) Mutikoek giltzak mahai gainean utzi dituzte.
boy-D.pl.erg keys table on leave aux

‘The children left the keys on the table.’

(103) Umeek puzzle bat egin dute.
kid-D.pl.erg puzzle one do aux
‘The children made a puzzle.’

(104) Gazte hauek beraien bikoteak maite dituzte.
young these.erg their couple love aux
‘These young people love their partners.’

(105) Bidaiari batzuk maletak daramatzate.
traveler some suitcase bring.prog
‘Some travelers carry suitcases.’

(106) Garbitzaileek lorontziak hautsi dituzte.
cleaner-D.pl.erg vases break aux
‘The cleaners broke the vases.’

(107) Gonbidatu hauek zukua edan dute.
guest these.erg juice drink aux
‘These guests drank juice.’

(108) Ume batzuk orriak margotu dituzte.
kid some.erg pages paint aux
‘Some children painted sheets.’

(109) Gizon bakoitzak kutxa bat bultzatzen du.
man each-D.sg.erg box one push.prog aux
‘Each man is pushing a box.’

(110) Gonbidatuek globoak puztutzen dituzte.
guest-D.pl.erg balloons inflate.prog aux
‘The guests are blowing up balloons.’

(111) Ume hauek orriak mozten dituzte.
kid these pages cut.prog aux
‘These children are cutting sheets.’

(112) Ikasleek notak hartzen dituzte.
student-D.pl.erg notes take.prog aux
‘The students are taking notes.’

(113) Neskek dorre bat eraiki dute.
girl-D.pl.erg tower one build aux
‘The girls built a tower.’

(114) Denak saltoka ari dira.
all-D.pl.erg jumping prog aux
‘Everybody is jumping.’

(115)	Langileek	beren	lana	egiten	dute.
	worker-D.pl.erg	their	work	do.prog	aux
	'The workers are doing their job.'				

(116)	Langileak	greban	daude.
	worker-D.pl.erg	strike.ines	aux
	'The workers are on strike.'		

(117)	Turistek	arrainak	arrantzatu	dituzte.
	tourist-D.pl.erg	fishes	catch	aux
	'The tourists have fished some fish.'			

(118)	Umeek	leihoak	itsi	dituzte.
	kid-D.pl.erg	windows	close	aux
	'The children have closed the windows.'			

(119)	Gonbidatuek	loreak	ekarri	dituzte.
	guest-D.pl.erg	flowers	bring	aux
	'The guests brought flowers.'			

(120)	Mutikoek	beren	gurasoak	jarraitzen	dituzte.
	boy-D.pl.erg	their	parents	follow.prog	aux
	'The boys are following their parents.'				

(121)	Neskak	gitarra	jotzen	du.
	girl-D.eg.erg	guitar	play.prog	aux
	'The girl plays the guitar.'			

(122)	Neskak	pianoa	jotzen	du.
	girl-D.eg.erg	piano	play.prog	aux
	'The girl plays the piano.'			

(123)	Emakumea	etzanda	dago.
	woman-D.sg	lay down	be.loc
	'The woman is lying down.'		

(124)	Mutila	soka	saltoan	ari	da.
	boy-D.sg	rope	jump.ines	prog	aux
	'The boy is skipping.'				

(125)	Neskak	hanka	hautsi	du.
	girl-D.sg.erg	leg	break	aux
	'The girl broke her leg.'			

(126)	Mutilak	besoa	hautsi	du.
	boy-D.sg.erg	arm	break	aux
	'The boy broke his arm.'			

(127)	Neskak	kutxa	altxatzen	du.
	girl-D.sg.erg	box	raise.prog	aux

‘The girl raises the box.’

(128) Langileak zulo bat egin du.
girl-D.sg.erg hole one do aux
‘The worker dug a hole.’

(129) Neskek txakur bakarra paseatu dute elkarrekin.
girl-D.pl.erg dog single walk aux together
‘The girls walked a single dog together.’

(130) Neskak txakur guztiei hezur bakarra eman die.
girl-D.sg.erg dog all-D.pl.dat bone single give aux
‘The girl gave a single bone to all the dogs.’

(131) Neskak txakur bati hezur guztiak eman dizkio.
girl-D.sg.erg dog one-dat bone all-D.pl give aux
‘The girl gave all the bones to a dog.’

(132) Arkatza liburuaren ondoan dago.
pencil-D.sg book-D.sg.gen beside is
‘The pencil is next to the book.’

(133) Gizon batek txapela dauka.
man one.erg hat-D.sg has
‘A man has a hat.’

(134) Foku bat jarri diote abeslari bakoitzari.
spotlight one put aux singer each-D.sg.dat
‘They put a spotlight to each singer.’

(135) Jonek ikasle bakoitzaren argazki bat dauka.
Jone.erg student each-D.sg.gen picture one has
‘Jone has a picture of each student.’

(136) Herriko festetan, ikurrina bat jarri zuten etxe
town.gen festival.in flag one put aux house
bakoitzaren aurrean.
each-D.sg.gen front.in
‘In the festival, they put a flag in front of each house.’

(137) Neskak txakur guztiak paseatu dute.
girl-D.sg.erg dog all-D.pl walk aux
‘The girl walked all the dogs.’

(138) Neska bakoitzak txakur bat paseatu du.
girl each-D.sg.erg dog one walk aux
‘Each girl walked a dog.’

(139) Jonek gizona kataloxekin ikusi du.
Jone.erg man-D.sg.abs telescope.with see aux

‘Jone saw the man with the binoculars.’

(140)

Aizkolari	bakoitzak	enbor	bat	moztu	du.
woodcutter	each-D.sg.erg	trunk	one	cut	aux

‘Each woodcutter cut a trunk.’

(141)

Mutikoak	espaloia	margotzen	du.
boy-D.sg.erg	sidewalk	paint	aux

‘The boy painted the sidewalk.’

(142)

Arkatza	liburuaren	gainean	dago.
pencil-D.sg	book-D.sg.gen	over.in	is

‘The pencil is on the book.’

(143)

Mutikoak	etxearen	aurrekaldea	margotzen	du.
boy-D.sg	house-D.sg.gen	façade	paint.prog	aux

‘The boy paints the façade of the house.’

(144)

Neskak	sei	sagar	ditu	eskuetan.
girl-D.sg.erg	six	apple	has	hands.in

‘The girls has six apples in her hands.’

B) SPANISH STIMULI – TEST
[used both for Castilian Spanish and Basque Country-Spanish]

DP-DP (partitive)

WITHOUT NO

(1) Ninguno de los estudiantes lee ninguno de los libros.
none of D.pl students has read none of D.pl books
'None of the students has read any of the books.'

(2) Ninguno de los estudiantes ha metido ninguna de las
none of D.pl students has introduced none of D.pl
cosas en la bolsa
things in the bag
'None of the students puts any of the things in the bag.'

(3) Ninguno de los jardineros ha recogido ninguna de las
none of D.pl gardeners has collect none of D.pl
hojas.
leaves
'None of the gardeners grasped any of the leaves.'

(4) Ninguno de los pescadores ha pescado ninguno de los
none of D.pl fishermen has fished none of D.pl
peces.
fishes
'None of the fishermen fished any of the fish.'

(5) Ninguno de los chicos ha golpeado ninguna de las
none of D.pl boys has hit none of D.pl
pelotas.
balls
'None of the boys hit any of the balls.'

(6) Ninguno de los músicos ha comprado ninguno de los
none of D.pl musicians has bought none of D.pl
instrumentos
instruments
'None of the musicians bought any of the instruments.'

WITH NO

(7) Ninguno de los campesinos no ha alimentado ninguna de
none of D.pl farmers not has fed none of
las vacas
D.pl cows
SN: 'None of the farmers fed any of the cows.'
DN: 'All the farmers fed the cows.'

(8) Ninguno de los cazadores no ha cazado ninguno de
none of D.pl hunters not has hunted none of

los pájaros.
D.pl birds
SN: ‘None of the hunters hunted any of the birds.’
DN: ‘All the hunters hunted the birds.’

(9) Ninguno de los niños no ha roto ninguna de las
none of D.pl kids not has broken none of D.pl
camisetas.
t-shirts
SN: ‘None of the kids tore any of the t-shirts.’
DN: ‘All the kids tore the t-shirts.’

(10) Ninguno de los niños no ha tocado ninguna de las
none of D.pl kids not has touched none of D.pl
flores.
flowers
SN: ‘None of the kids touched any of the flowers.’
DN: ‘All the kids touched the flowers.’

(11) Ninguno de los chicos no recogió ninguna de las
none of D.pl boys not collected none of D.pl
cosas.
things
SN: ‘None of the boys collected any of the things.’
DN: ‘All the boys collected the things.’

(12) Ninguno de los chicos no construyó ninguna de las
none of D.pl boys not built none of D.pl
torres.
towers
SN: ‘None of the boys built any of the towers.’
DN: ‘All the boys built the towers.’

Pro-Pro

WITHOUT NO

(13) Nadie ha roto nada.
nobody has broken nothing/anything
‘Nobody broke anything.’

(14) Nadie ha tocado nada.
nobody has touched nothing/anything
‘Nobody touched anything.’

(15) Nadie ha recogido nada
nobody has collected nothing/anything
‘Nobody collected anything.’

(16) Nadie ha visto a nadie.
nobody has seen to nobody/anybody
‘Nobody saw anybody.’

(17) Nadie tira nada.
nobody throw nothing/anything
'Nobody is throwing anything.'

(18) Nadie lee nada
nobody read nothing/anything
'Nobody is reading anything.'

WITH NO

(19) Nadie no ordena nada.
nobody no order nothing/anything
SN: 'Nobody is ordering anything.'
DN: 'Everybody is ordering something.'

(20) Nadie no construye nada.
nobody no build nothing/anything
SN: 'Nobody is building anything.'
DN: 'Everybody is building something.'

(21) Nadie no come nada.
nobody no eat nothing/anything
SN: 'Nobody is eating anything.'
DN: 'Everybody is eating something.'

(22) Nadie no toca nada. (instrumento)
nobody no touch nothing/anything
SN: 'Nobody is playing anything.'
DN: 'Everybody is playing something.'

(23) Nadie no alquila nada.
nobody no rent nothing/anything
SN: 'Nobody rents anything.'
DN: 'Everybody rents something.'

(24) Nadie no enciende nada.
nobody no light nothing/anything
SN: 'Nobody lights up anything.'
DN: 'Everybody lights up something.'

Pro-DP

WITHOUT NO

(25) Nadie canta ninguna de las canciones.
nobody sing none of D.pl songs
'Nobody is singing any of the songs.'

(26) Nadie pega ninguna de las piezas
nobody glue none of D.pl pieces
'Nobody is gluing the pieces.'

(27) Nadie alquila ninguno de los coches.
nobody rent none of D.pl cars
'Nobody is renting any of the cars.'

(28) Nadie toca ninguna de las obras de arte.
nobody touch none of D.pl work of art
'Nobody is touching any of the artworks.'

(29) Nadie enciende ninguna de las velas.
nobody light none of D.pl candles
'Nobody is lightening up any of the candles.'

(30) Nadie ha traído ninguno de los regalos.
nobody has brougt none of D.pl presents
'Nobody brought any of the presents.'

WITH NO

(31) Nadie no lleva ninguna de las maletas.
nobody not carry none of D.pl suitcases
SN: 'Nobody is carrying any of the suitcases.'
DN: 'Everybody is carrying the suitcases.'

(32) Nadie no mueve ninguna de las cajas.
nobody not move none of D.pl boxes
SN: 'Nobody is moving any of the boxes.'
DN: 'Everybody is moving the boxes.'

(33) Nadie no recoge ninguno de los libros.
nobody not collect none of D.pl books
SN: 'Nobody collects any of the books.'
DN: 'Everybody collects the books.'

(34) Nadie no construye ninguna de las torres.
nobody not build none of D.pl towers
SN: 'Nobody builds any of the towers.'
DN: 'Everybody builds the towers.'

(35) Nadie no lee ninguno de los libros.
nobody not read none of D.pl books
SN: 'Nobody is reading any of the books.'
DN: 'Everybody is reading the books.'

(36) Nadie no tira ninguno de los floreros.
nobody not throw none of D.pl vases
SN: 'Nobody throws any of the vases.'
DN: 'Nobody throws the vases.'

DP-Pro

WITHOUT NO

(37)

Ninguno	de	los	jugadores	lanza	nada.
none	of	D.pl	players	throw	nothing/anything

‘None of the players is throwing anything.’

(38)

Ninguno	de	los	invitados	come	nada.
none	of	D.pl	guests	eat	nothing/anything

‘None of the guests is eating anything.’

(39)

Ninguno	de	los	niños	bebe	nada.
none	of	D.pl	kids	drink	nothing/anything

‘None of the children is drinking anything.’

(40)

Ninguno	de	los	estudiantes	escribe	nada.
none	of	D.pl	students	write	nothing/anything

‘None of the strudents is writing anything.’

(41)

Ninguno	de	los	visitantes	roba	nada.
none	of	D.pl	guests	throw	nothing/anything

‘None of the visitors is stealing anything.’

(42)

Ninguna	de	las	costureras	enreda	nada.
none	of	D.pl	tailors	tangle	nothing/anything

‘None of the tailors is tangling anything.’

WITH NO

(43)

Ninguno	de	los	niños	no	levanta	nada.
none	of	D.pl	kids	not	lift	nothing/anything

SN: ‘None of the children is lifting anything.’
DN: ‘All the children are lifting something.’

(44)

Ninguno	de	los	invitados	no	ha	traído	nada
none	of	D.pl	guests	not	has	bring	nothing/anything

SN: ‘None of the guests brought anything.’
DN: ‘All the guests brought something.’

(45)

Ninguno	de	los	niños	no	construye	nada.
none	of	D.pl	kids	not	build	nothing/anything

SN: ‘None of the children is building anything.’
DN: ‘All the children are building something.’

(46)

Ninguno	de	los	estudiantes	no	lleva	nada.
none	of	D.pl	students	not	carry	nothing/anything

SN: ‘None of the students is carrying anything.’
DN: ‘All the students are carrying something.’

(47)

Ninguno	de	los	estudiantes	no	enciende	nada.
none	of	D.pl	students	not	light	nothing/anything

SN: ‘None of the students lights up anything.’
DN: ‘All the students light up something.’

(48) Ninguno de los invitados no recoge nada.
none of D.pl guests not carry nothing/anything
SN: ‘None of the guests tidies up anything.’
DN: ‘All the guests tidy up something.’

CONTROL DOUBLE NEGATION

(49) No es verdad que los trabajadores no hayan enterrado nada.
not is true that D.pl workers not have buried nothing
‘It is not true that the workers have not buried anything.’

(50) No es verdad que los pescadores no hayan pescado nada.
not is true that D.pl fishermen not have fished nothing
‘It is not true that the fishermen have not fished anything.’

(51) No es verdad que ningún alumno haya votado.
not is true that none student have voted
‘It is not true that no student has voted.’

(52) No es verdad que ningún invitado haya comido.
not is true that none guest have eaten
‘It is not true that no guest has eaten.’

(53) No es verdad que ningún niño levante nada.
not is true that none kid lift nothing/anything
‘It is not true that no child lifted anything.’

(54) No es verdad que ningún niño haya juntado ninguna pieza.
not is true that none kid have joined none/any piece
‘It is not true that no child joined any piece.’

(55) No es verdad que nadie haya abierto la ventana.
not is true that nobody have open D.sg window
‘It is not true that nobody opened the window.’

(56) No es verdad que nadie haya comprado una flor.
not is true that nobody have bought one flower
‘It is not true that nobody bought a flower.’

(57) No es verdad que nadie haya recogido un libro.
not is true that nobody have collected one book
‘It is not true that nobody collects a book.’

(58) No es verdad que nadie haya encendido una vela.
not is true that nobody have lit one candle
‘It is not true that nobody lights up a candle.’

(59) No es verdad que nadie haya bebido vino.
not is true that nobody have drunk wine
‘It is not true that nobody drinks wine.’

(60) No es verdad que nadie haya comido un dulce.

not is true that nobody have eaten one sweet
'It is not true that nobody eats a sweet.'

CONTROL SINGLE NEGATION SUBJECT

(61) Nadie sigue al primer chico.
nobody follow D.sg.dat first boy
'Nobody is following the first child.'

(62) Ningún visitante mira una joya.
none visitor look one jewel
'No visitor is looking at any jewel.'

(63) Nadie ha perdido las llaves.
nobody has lost D.pl keys
'Nobody lost the keys.'

(64) Ningún trabajador hace un agujero.
none worker make one hole
'No worker is making a hole.'

(65) Nadie mete las cosas de la escuela en la bolsa.
none put D.pl things of D.sg school in D.sg bag
'Nobody puts the school things in the bag.'

(66) Ninguna niña corta un papel con las tijeras.
none girl cut one paper with D.pl scissors
'No child is cutting a paper with the scissors.'

(67) Nadie ha traído las maletas.
nobody has brought D.pl suitcases
'Nobody brought the suitcases.'

(68) Ningún estudiante tiene una hoja pintada.
none student has one sheet painted
'No student has a painted sheet.'

(69) Nadie come los dulces.
nobody eat D.pl sweets
'Nobody eats the sweets.'

(70) Ningún estudiante rompe su camiseta.
none student break his/her t-shirt
'No student tears his t-shirt.'

(71) Nadie tira un florero.
nobody throw one vase
'Nobody throws a vase.'

(72) Ningún niño bebe el vino.
none kid drink D.sg wine
'No kid drinks wine.'

CONTROL SINGLE NEGATION OBJECT

(73) Los trabajadores no hacen ningún agujero.
D.pl workers not make none hole
'The workers don't make any hole.'

(74) Los pescadores no han pescado nada.
D.pl fishermen not have fished nothing/anything
'The fishermen didn't fish anything.'

(75) Los turistas no han perdido ninguna maleta.
D.pl tourists not have lost none suitcase
'Tourists didn't lose any suitcase.'

(76) Los estudiantes no han pintado ningún papel.
D.pl students not have painted none sheet
'The students didn't paint any sheet.'

(77) Los invitados no trajeron ningún ramo de flores.
D.pl guests not brought none bouquet of flowers
'The guests didn't bring any flowers.'

(78) Los niños no han ordenado ningún libro.
D.pl kids not have ordered none book
'The children didn't collect any book.'

(79) Los niños no encendieron ninguna vela.
D.pl kids not lit none candle
'The children didn't light up any candle.

(80) El jardinero no recogió ninguna hoja.
D.pl gardener not collected none leave
'The garderer didn't rak any leave.'

(81) El chico no come ningún pastel.
D.sg boy not eat none sweet
'The boy doesn't eat any sweet.'

(82) El visitante no toca ningún cuadro.
D.sg visitor not touch none painting
'The visitor doesn't touch any painting.'

(83) La chica no bebe ningún vino.
D.sg girl not drink none wine
'The girl didn't drink any wine.'

(84) El estudiante no enciende ninguna vela.
D.sg sudent not light none candle
'The student doesn't light up any candle.'

CONTROL UNIVERSAL READING

(85) Todos los alumnos escriben algo.
all D.pl students write something
'All students are writing something.'

(86) Jon toca todos los objetos.
Jon touch all D.pl objects
'Jon is touching the objects.'

(87) Todos han traído algo.
all have brought something
'Everybody brought something.'

(88) Ane mueve todas las cosas.
Ane move all D.pl things
'Ane is moving all the things.'

(89) Los niños pintan todas las hojas.
D.pl kids paint all D.pl sheets
'The children are painting all the sheets.'

(90) Todos han dado un voto.
all have given one vote
'Everybody gave their vote.'

(91) Los trabajadores han hecho todos los agujeros.
D.pl workers have done all D.pl holes
'The workers made all the holes.'

(92) Cada trabajador hace su trabajo.
each worker make his/her job
'Each worker did his job.'

(93) Todos han bebido algo.
all have drunk something
'Everybody drank something.'

(94) Ane ha pintado todos los cuadros.
Ane has painted all D.pl paintings
'Ane has painted all the paintings.'

(95) Todos los estudiantes encienden algo.
all D.pl students light something
'All the students light up something.'

(96) Jone mete todas las cosas en la bolsa.
Jone put all D.pl things in D.sg bag
'Jone puts all the things in the bag.'

FILLERS

(97) Los botánicos investigan plantas.

D.pl botanists investigate plants
‘Botanists investigate plants.’

(98) Los niños ven una serie.
D.pl kids see one series
‘The children are watching a series.’

(99) Los estudiantes leen algo.
D.pl students read something
‘The students are reading something.’

(100) Cada alumno hace una actividad diferente.
each student make one activity different
‘Each student is doing a different activity.’

(101) Los votantes están votando.
D.pl voters be.loc voting
‘Voters are voting.’

(102) Los chicos han dejado las llaves encima de la mesa.
D.pl boys have left D.pl keys over of D.sg table
‘The children left the keys on the table.’

(103) Los niños han hecho un puzzle.
D.pl kids have made one puzzle
‘The children made a puzzle.’

(104) Estos jóvenes aman a sus parejas.
Dem.pl young love to their partners
‘These young people love their partners.’

(105) Algunos viajeros llevan maletas.
Some traveler carry suitcases
‘Some travelers carry suitcases.’

(106) Los limpiadores han roto los jarrones.
D.pl cleaners have broken D.pl vases
‘The cleaners broke the vases.’

(107) Estos invitados han bebido zumo.
Dem.pl guests have drunk juice
‘These guests drank juice.’

(108) Unos niños han pintado hojas.
some kids have painted sheets
‘Some children painted sheets.’

(109) Cada hombre empuja una caja.
each man push one box
‘Each man is pushing a box.’

(110) Los invitados inflan globos.

D.pl guests inflate balloons
'The guests are blowing up balloons.'

(111) Estos niños corta hojas.
Dem.pl kids cut sheets
'These children are cutting sheets.'

(112) Los estudiantes toman notas.
Dem.pl students take notes
'The students are taking notes.'

(113) Las chicas han construido una torre.
D.pl girls have built one tower
'The girls built a tower.'

(114) Todos saltan.
all jump
'Everybody is jumping.'

(115) Los trabajadores hacen su trabajo.
D.pl workers do his/her job
'The workers are doing their job.'

(116) Los trabajadores hacen huelga.
D.pl workers make strike
'The workers are on strike.'

(117) Los turistas han pescado peces.
D.pl tourists have fished fishes
'The tourists have fished some fish.'

(118) Los niños han cerrado las ventanas.
D.pl kids have closed D.pl windows
'The children have closed the windows.'

(119) Los invitados han traído flores.
D.pl guests have brought flowers
'The guests brought flowers.'

(120) Los chicos siguen a sus padres.
D.pl boys follow to their parents
'The boys are following their parents.'

(121) La chica toca la guitarra.
D.sg girl touch D.sg guitar
'The girl plays the guitar.'

(122) La chica toca el piano.
D.sg girl touch D.sg piano
'The girl plays the piano.'

(123) La mujer está tumbada.

D.sg woman be.loc laying down
'The woman is lying down.'

(124) El chico salta a la comba.
D.sg boy jump to D.sg skip rope
'The boy is skipping.'

(125) La chica se ha roto la pierna.
D.sg girl se has broken D.sg leg
'The girl broke her leg.'

(126) El chico se ha roto el brazo.
D.sg boy se has broken D.sg arm
'The boy broke his arm.'

(127) La chica levanta la caja.
D.sg girl lift D.sg box
'The girl lifts the box.'

(128) El trabajador ha hecho un agujero.
D.sg worker has made one hole
'The worker dug a hole.'

(129) Las chicas han paseado un perro juntas.
D.pl girls have walked one dog together
'The girls walked a dog together.'

(130) La chica ha dado un único hueso a todos los perros.
D.sg girl has given one single bone to all D.pl dogs
'The girl gave a single bone to all the dogs.'

(131) La chica ha dado todos los huesos a un perro.
D.sg girl has given all D.pl bones to one dog
'The girl gave all the bones to a dog.'

(132) El lapiz está junto al libro.
D.sg pencil be.loc beside to.D.sg book
'The pencil is next to the book.'

(133) Un hombre lleva boina.
one man bring beret
'A man is wearing a beret.'

(134) Han puesto un foco a cada cantante.
have put one spotlight to each singer
'They put a spotlight to each singer.'

(135) Jone tiene una foto de cada estudiante.
Jone has one picture of each student
'Jone has a picture of each student.'

(136) En fiestas, pusieron una bandera en frente de cada

in festivals put one flag in front of each
casa.
house
'In the festival, they put a flag in front of each house.'

(137) La chica ha paseado a todos los perros.
D.sg girl has waled to all D.pl dogs
'The girl walked all the dogs'.

(138) Cada chica ha paseado un perro.
each girl has waled one dog
'Each girl walked a dog.'

(139) Jone ha visto al hombre con catalejos.
Jone has seen to.D.sg man with binoculars
'Jone saw the man with the binoculars.'

(140) Cada aizkolari ha cortado un tronco.
each woodcutter has cut one trunk
'Each woodcutter cut a trunk.'

(141) El chico pinta la acera.
D.sg boy paint D.sg pavement
'The boy paints the pavement.'

(142) El lapiz está encima del libro.
D.sg pencil be.loc over of.D.sg book
'The pencil is over the book.'

(143) El chico pinta la fachada de la casa.
D.sg boy paint D.sg façade of D.sg house
'The boy paints the façade of the house.'

(144) La chica tiene seis manzanas en las manos.
D.sg girl has six apples in D.pl hands
'The girls has six apples in her hands.'

Appendix 2. Demographic information about the participants of each linguistic group. Cities of birth and cities of residence also include the region or state in which they are located, where the following abbreviations have been used: A[raba], B[izkaia], G[ipuzkoa], M[adrid], and N[afarroa]. BC-Spanish participants, who are native/near-native of Basque, claimed to use more than 50% of Spanish in their daily life.

Basque group

User ID	Age	Gender	Native city	Living city	Knowledge of Basque
01	19	M	Beasain (G)	=	native
02	18	F	Usurbil (G)	Vitoria (A)	native
03	18	F	Vitoria (A)	=	near-native
04	18	F	Beasain (G)	Vitoria (A)	native
05	18	F	Sopela (B)	Vitoria (A)	native
06	18	F	Irun (G)	Vitoria (A)	near-native
07	19	F	Irun (G)	Vitoria (A)	native
08	19	F	Lezo (G)	=	native
09	18	F	Durango (B)	Vitoria (A)	native
10	18	F	Zarautz (G)	Vitoria (A)	native
11	20	F	Burlata (N)	Vitoria (A)	near-native
12	18	F	Bera (N)	=	native
13	18	M	Ondarroa (B)	Vitoria (A)	native
14	18	F	Amurrio (A)	Vitoria (A)	native
15	21	F	Donostia (G)	=	native
16	18	M	Zarautz (G)	Vitoria (A)	native
17	18	F	Zarautz (G)	Vitoria (A)	native
18	18	F	Irun (G)	Vitoria (A)	native
19	18	F	Forua (B)	=	native
20	22	F	Bilbo (B)	Vitoria (A)	native
21	18	F	Donostia (G)	=	native
22	18	M	Villabona (G)	=	native
23	19	M	Urnieta (G)	Vitoria (A)	native
24	18	M	Algorta (B)	=	native
25	19	M	Zubieta (N)	Vitoria (A)	native
26	20	M	Durango (B)	Vitoria (A)	native
27	18	M	Bergara (G)	=	native
28	18	F	Azkoitia (G)	Vitoria (A)	native
29	19	F	Oiz (N)	Vitoria (A)	native
30	18	M	Hondarribia (G)	Vitoria (A)	native
31	19	F	Ordizia (G)	=	near-native
32	18	M	Arrigorriaga (B)	=	native
33	18	F	Vitoria (A)	=	native

BC-Spanish group

User ID	Age	Gender	Native city	Living city	Knowledge of Basque
01	20	M	Larrabetzu (B)	=	native
02	21	F	Urnieta (G)	Vitoria (A)	near-native
03	20	M	Alsasua (N)	=	native
04	20	F	Iruña (N)	Vitoria (A)	native
05	20	F	Orereta (G)	=	native
06	20	F	Zizurkil (G)	Vitoria (A)	native
07	20	F	Vitoria (A)	=	native
08	22	F	Bilbo (B)	Vitoria (A)	native
09	21	M	Iruña (N)	=	near-native
10	20	M	Ortuella (B)	Vitoria (A)	native
11	20	M	Galdakao (B)	=	near-native
12	22	M	Araia (A)	=	native
13	20	M	Irun (G)	Vitoria (A)	native
14	20	F	Zumarraga (G)	=	native
15	20	F	Beasain (G)	=	native
16	20	M	Urnieta (G)	Vitoria (A)	native
17	20	F	Arrigorriaga (B)	=	native
18	20	M	Zarautz (G)	Vitoria (A)	native
19	20	F	Bilbo (B)	=	native
20	21	M	Vitoria (A)	=	native
21	21	F	Orio (G)	Vitoria (A)	native
22	20	F	Vitoria (A)	=	native
23	21	F	Urnieta (G)	=	native
24	20	F	Zarautz (G)	Vitoria (A)	near-native
25	20	M	Forua (B)	Vitoria (A)	native
26	20	M	Irun (G)	Vitoria (A)	native
27	20	F	Irurtzun (N)	Vitoria (A)	near-native
28	20	F	Vitoria (A)	=	native
29	20	F	Tolosa (G)	=	native
30	20	M	Zarautz (G)	Vitoria (A)	near-native
31	25	F	Iruña (N)	=	near-native
32	20	F	Lekeitio (B)	Vitoria (A)	near-native
33	20	M	Itziar (G)	Vitoria (A)	near-native
34	20	M	Zarautz (G)	Vitoria (A)	near-native
35	21	F	Lesaka (N)	Vitoria (A)	native
36	20	M	Alsasua (N)	=	near-native
37	20	M	Vitoria (A)	=	native
38	20	M	Santurtzi (B)	=	native

Castilian-Spanish group

User ID	Age	Gender	Native city	Living city	Knowledge of Spanish
01	19	F	Gothemburg (Sweden)	Alcalá H. (M)	near-native
02	19	F	Madrid (M)	=	native
03	19	F	Alcalá H. (M)	=	native
04	19	F	Alcalá H. (M)	Madrid (M)	native
05	22	M	(Romania)	Alcalá H. (M)	native
06	21	F	Bordeaux (France)	Alcalá H. (M)	near-native
07	19	F	Getafe (M)	=	native
08	19	F	Madrid (M)	=	native
09	24	F	Alcalá H. (M)	=	native
10	21	F	Kardzhali (Bulgaria)	Alcalá H. (M)	near-native
11	19	F	Torrejón A. (M)	=	native
12	25	F	Alcalá H. (M)	Pioz (Gua)	native
13	19	F	Madrid (M)	Rivas (M)	native
14	20	F	Barcelona (Barcelona)	Madrid (M)	native
15	21	F	Algete (M)	=	native
16	20	F	Alcalá H. (M)	=	native
17	19	F	Coslada (M)	=	near-native
18	19	F	Madrid (M)	Daganzo A. (M)	native
19	23	F	Alcalá H. (M)	=	native
20	24	F	Madrid (M)	=	native
21	19	M	Madrid (M)	=	native
22	19	F	Alcalá H. (M)	=	native
23	20	F	Shanghai (China)	Alcalá H. (M)	near-native
24	19	F	Madrid (M)	Belmonte T. (M)	native
25	20	F	Shanghai (China)	Alcalá H. (M)	near-native
26	19	M	Torrejón A. (M)	=	native
27	20	F	Torrejón A. (M)	=	native
28	20	F	Madrid (M)	=	native
29	51	F	Madrid (M)	Alcalá H. (M)	native
30	26	M	Torrejón A. (M)	Alcalá H. (M)	native

Appendix 3. Descriptive results for Acceptability, ZAcceptability and Interpretation of controls, fillers and critical stimuli in Basque, BC-Spanish and Castilian Spanish

Table 1. Acceptability and Interpretation of controls and fillers

				Acceptability		ZAcceptability		SN Interpretation	
Group	Type	Subtype	N	M	SD	M	SD	M	SD
Basque	Control	SNS	396	4.402	.982	.202	.695	.949	.219
		SNO	396	4.614	.811	.370	.552	.932	.252
		Universal Reading	396	4.705	.702	.429	.517	.980	.141
		Double Negation	396	4.093	1.093	-.043	.774	.947	.224
		**Total**	**1584**	**4.453**	**.939**	**.239**	**.668**	**.952**	**.214**
	Filler		1584	4.722	.713	.449	.530	.984	.127
BC-Spa	Control	SNS	450	4.669	.784	.269	.605	.942	.234
		SNO	453	4.711	.824	.317	.497	.881	.324
		Universal Reading	455	4.875	.450	.437	.307	.982	.132
		Double Negation	456	4.454	.982	.085	.709	.840	.367
		**Total**	**1814**	**4.677**	**.798**	**.277**	**.564**	**.911**	**.284**
	Filler		1814	4.872	.484	.436	.357	.992	.091
Cast-Spa	Control	SNS	360	4.547	.898	.275	.696	.944	.229
		SNO	360	4.564	.832	.262	.682	.953	.212
		Universal Reading	360	4.758	.684	.464	.543	.981	.138
		Double Negation	359	3.989	1.251	-.196	.898	.838	.369
		**Total**	**1439**	**4.465**	**.981**	**.202**	**.755**	**.929**	**.257**
	Filler		1439	4.760	.643	.454	.544	.983	.128

Table 2. Acceptability and Interpretation for criticals

				Acceptability		ZAcceptability		SN Interpretation	
Group	Neg Marker	Structure	N	M	SD	M	SD	M	SD
Basque	Without *ez*	DP-DP	198	1.874	1.266	-1.725	.969	.697	.461
		DP-Pro	198	2.611	1.523	-1.135	1.126	.934	.248
		Pro-DP	198	1.737	1.167	-1.837	.924	.778	.417
		Pro-Pro	198	2.818	1.721	-.987	1.324	.949	.220
		**Total**	**792**	**2.260**	**1.506**	**-1.421**	**1.154**	**.840**	**.367**
	With *ez*	DP-DP	198	3.778	1.214	-.295	.931	.960	.197
		DP-Pro	198	4.237	.997	.068	.696	.889	.315
		Pro-DP	198	4.086	1.165	-.035	.797	.970	.172
		Pro-Pro	198	4.697	.747	.440	.533	.980	.141
		**Total**	**792**	**4.199**	**1.096**	**.044**	**.797**	**.949**	**.219**

BC-Spa	Without *no*	DP-DP	227	3.956	1.432	-.294	.985	.969	.173
		DP-Pro	227	4.463	1.110	.114	.758	.885	.319
		Pro-DP	226	4.469	1.055	.064	1.134	.929	.257
		Pro-Pro	227	4.696	.781	.310	.439	.952	.215
		**Total**	**907**	**4.396**	**1.149**	**.049**	**.895**	**.934**	**.249**
	With *no*	DP-DP	225	2.404	1.593	-1.554	1.236	.573	.496
		DP-Pro	228	2.579	1.620	-1.414	1.182	.531	.500
		Pro-DP	227	2.423	1.615	-1.533	1.188	.573	.496
		Pro-Pro	227	2.648	1.703	-1.396	1.381	.533	.500
		**Total**	**907**	**2.514**	**1.634**	**-1.474**	**1.249**	**.552**	**.498**
Cast-Spa	Without *no*	DP-DP	180	3.633	1.410	-.519	1.138	.861	.347
		DP-Pro	180	4.194	1.149	-.033	.829	.800	.401
		Pro-DP	180	4.278	1.036	-.007	.833	.878	.328
		Pro-Pro	180	4.500	.954	.224	.756	.856	.353
		**Total**	**720**	**4.151**	**1.191**	**-.083**	**.939**	**.849**	**.359**
	With *no*	DP-DP	180	2.683	1.470	-1.256	1.054	.483	.501
		DP-Pro	180	2.767	1.387	-1.196	1.114	.400	.491
		Pro-DP	180	2.594	1.534	-1.292	1.241	.444	.498
		Pro-Pro	180	2.783	1.525	-1.164	1.218	.456	.499
		**Total**	**720**	**2.707**	**1.479**	**-1.227**	**1.158**	**.446**	**.497**

Table 3. Relation between Acceptability and Interpretation in criticals

				Acceptability		ZAcceptability	
Group	Neg Marker	Interpretation	N	M	SD	M	SD
Basque	Without *ez*	DN	127	1.858	1.233	-1.675	.955
		SN	665	2.337	1.542	-1.373	1.183
	With *ez*	DN	40	4.200	1.018	.033	.742
		SN	752	4.199	1.101	.045	.800
BC-Spa	Without *no*	DN	60	4.150	1.338	-.051	.900
		SN	847	4.413	1.134	.056	.895
	With *no*	DN	406	2.648	1.562	-1.477	1.240
		SN	501	2.405	1.683	-1.472	1.258
Cast-Spa	Without *no*	DN	109	3.633	1.274	-.395	.991
		SN	611	4.244	1.153	-.028	.919
	With *no*	DN	399	2.684	1.371	-1.261	1.043
		SN	321	2.735	1.605	-1.185	1.288

Appendix 4. GLMMs and pairwise contrasts outputs

Model 1. Acceptability

Fixed Effects

Source	F	df1	df2	Sig.
Corrected Model	165.047	5	4784	.000
Group	.320	2	4784	.727
NegMarker	68.164	1	4784	.000
Group * NegMarker	377.175	2	4784	.000

Probability distribution: Normal

Link function: Identity

a. Target: ZAcceptability

Fixed Coefficients

Model Term	Coefficient	Std. Error	t	Sig.	95% CI Lower	95% CI Upper
Intercept	-1.421	.0652	-21.782	.000	-1.549	-1.293
Group=3	1.338	.0934	14.330	.000	1.155	1.521
Group=2	1.471	.0911	16.142	.000	1.292	1.649
Group=1	0[b]	.	.	.	.	.
NegMarker=1	1.465	.0840	17.445	.000	1.301	1.630
NegMarker=0	0[b]	.	.	.	.	.
[Group=3]*[NegMarker=1]	-2.609	.1199	-21.766	.000	-2.844	-2.374
[Group=3]*[NegMarker=0]	0[b]	.	.	.	.	.
[Group=2]*[NegMarker=1]	-2.988	.1177	-25.392	.000	-3.219	-2.757
[Group=2]*[NegMarker=0]	0[b]	.	.	.	.	.
[Group=1]*[NegMarker=1]	0[b]	.	.	.	.	.
[Group=1]*[NegMarker=0]	0[b]	.	.	.	.	.

Probability distribution: Normal

Link function: Identity

a. Target: ZAcceptability

b. This coefficient is set to zero because it is redundant.

Pairwise Contrasts

Group Pairwise Contrasts	Contrast Estimate	Std. Error	t	df	Adj. Sig.	95% CI Lower	95% CI Upper
Basque - BC-Spa	.023	.070	.334	4784	1.000	-.122	.168
Basque - Cast-Spa	-.033	.072	-.462	4784	1.000	-.186	.120
BC-Spa - Cast-Spa	-.056	.071	-.798	4784	1.000	-.225	.113

The sequential Bonferroni adjusted significance level is .05.

Confidence interval bounds are approximate.

Pairwise Contrasts

						95% CI	
Neg Marker Pairwise Contrasts	Contrast Estimate	Std. Error	t	df	Adj. Sig.	Lower	Upper
Without 'ez'/'no' - With 'ez'/'no'	.400	.048	8.256	4784	2.220E-16	.305	.495

The sequential Bonferroni adjusted significance level is .05.

Confidence interval bounds are approximate.

Pairwise Contrasts

							95% CI	
Neg Marker	Group Pairwise Contrasts	Contrast Estimate	Std. Error	t	df	Adj. Sig.	Lower	Upper
With 'ez'/'no'	Basque - BC-Spa	1.517	.091	16.652	4784	.000	1.313	1.722
	Basque - Cast-Spa	1.272	.093	13.622	4784	.000	1.048	1.495
	BC-Spa - Cast-Spa	-.246	.092	-2.664	4784	.008	-.426	-.065
Without 'ez'/'no'	Basque - BC-Spa	-1.471	.091	-16.142	4784	.000	-1.675	-1.267
	Basque - Cast-Spa	-1.338	.093	-14.330	4784	.000	-1.561	-1.114
	BC-Spa - Cast-Spa	.133	.092	1.443	4784	.149	-.048	.314

The sequential Bonferroni adjusted significance level is .05.

Confidence interval bounds are approximate.

Pairwise Contrasts

							95% CI	
Group	Neg Marker Pairwise Contrasts	Contrast Estimate	Std. Error	t	df	Adj. Sig.	Lower	Upper
Cast-Spa	Without 'ez'/'no' - With 'ez'/'no'	1.144	.086	13.375	4784	.000	.976	1.312
BC-Spa	Without 'ez'/'no' - With 'ez'/'no'	1.523	.082	18.476	4784	.000	1.361	1.684
Basque	Without 'ez'/'no' - With 'ez'/'no'	-1.465	.084	-17.445	4784	.000	-1.630	-1.301

The sequential Bonferroni adjusted significance level is .05.

Confidence interval bounds are approximate.

Model 2. Interpretation

Fixed Effects

Source	F	df1	df2	Sig.
Corrected Model	71.464	5	4832	.000
Group	12.457	2	4832	.000
NegMarker	100.389	1	4832	.000
Group * NegMarker	96.002	2	4832	.000

Probability distribution: Binomial

Link function: Logit

a. Target: SN Interpretation

Fixed Coefficients

					95% CI	Exp(Coefficient)	95% CI for Exp(Coefficient)		
Model Term	Coefficient	Std. Error	t	Sig.	Lower	Upper		Lower	Upper
Intercept	2.021	.2656	7.609	.000	1.500	2.542	7.548	4.484	12.705
Group=3	.113	.3822	.296	.767	-.636	.863	1.120	.529	2.369

Group=2	1.236	.3803	3.250	.001	.490	1.982	3.442	1.633	7.255
Group=1	0[b]	.	.	.	.	.	.	.	.
NegMarker=1	1.373	.2497	5.500	.000	.884	1.863	3.948	2.420	6.442
NegMarker=0	0[b]	.	.	.	.	.	.	.	.
[Group=3]*[NegMarker=1]	-3.764	.3264	-11.533	.000	-4.404	-3.125	.023	.012	.044
[Group=3]*[NegMarker=0]	0[b]	.	.	.	.	.	.	.	.
[Group=2]*[NegMarker=1]	-4.332	.3367	-12.868	.000	-4.992	-3.672	.013	.007	.025
[Group=2]*[NegMarker=0]	0[b]	.	.	.	.	.	.	.	.
[Group=1]*[NegMarker=1]	0[b]	.	.	.	.	.	.	.	.
[Group=1]*[NegMarker=0]	0[b]	.	.	.	.	.	.	.	.

Probability distribution: Binomial

Link function: Logit

a. Target: SN Interpretation

b. This coefficient is set to zero because it is redundant.

Pairwise Contrasts

Group Pairwise Contrasts	Contrast Estimate	Std. Error	t	df	Adj. Sig.	95% CI Lower	95% CI Upper
Basque - BC-Spa	.082	.032	2.560	4832	.021	.010	.154
Basque - Cast-Spa	.219	.052	4.201	4832	.000	.094	.343
BC-Spa - Cast-Spa	.137	.057	2.380	4832	.021	.019	.254

The sequential Bonferroni adjusted significance level is .05.

Confidence interval bounds are approximate.

Pairwise Contrasts

Neg Marker Pairwise Contrasts	Contrast Estimate	Std. Error	t	df	Adj. Sig.	95% CI Lower	95% CI Upper
Without 'ez'/'no' - With 'ez'/'no'	.163	.023	7.156	4832	9.568E-13	.119	.208

The sequential Bonferroni adjusted significance level is .05.

Confidence interval bounds are approximate.

Pairwise Contrasts

Neg Marker	Group Pairwise Contrasts	Contrast Estimate	Std. Error	t	df	Adj. Sig.	95% CI Lower	95% CI Upper
With 'ez'/'no'	Basque - BC-Spa	.394	.059	6.672	4832	.000	.261	.526
	Basque - Cast-Spa	.531	.065	8.181	4832	.000	.376	.687
	BC-Spa - Cast-Spa	.138	.087	1.590	4832	.112	-.032	.308
Without 'ez'/'no'	Basque - BC-Spa	-.080	.029	-2.745	4832	.018	-.150	-.010
	Basque - Cast-Spa	-.011	.038	-.296	4832	.767	-.085	.063
	BC-Spa - Cast-Spa	.069	.028	2.476	4832	.027	.006	.131

The sequential Bonferroni adjusted significance level is .05.

Confidence interval bounds are approximate.

Pairwise Contrasts

Group	Neg Marker Pairwise Contrasts	Contrast Estimate	Std. Error	t	df	Adj. Sig.	95% CI Lower	95% CI Upper
Cast-Spa	Without 'ez'/'no' - With 'ez'/'no'	.458	.050	9.183	4832	.000	.360	.556

BC-Spa	Without 'ez'/'no' - With 'ez'/'no'	.389	.053	7.366	4832	.000	.285	.492
Basque	Without 'ez'/'no' - With 'ez'/'no'	-.085	.023	-3.697	4832	.000	-.129	-.040

The sequential Bonferroni adjusted significance level is .05.

Confidence interval bounds are approximate.

Model 3. Correlation between interpretation and acceptability

Fixed Effects

Source	F	df1	df2	Sig.
Corrected Model	77.618	11	4778	.000
Group	.047	2	4778	.954
NegMarker	27.692	1	4778	.000
Respuesta_imagen	6.960	1	4778	.008
Group * NegMarker	203.604	2	4778	.000
Group * Respuesta_imagen	1.074	2	4778	.342
NegMarker * Respuesta_imagen	4.610	1	4778	.032
Group * NegMarker * Respuesta_imagen	.958	2	4778	.384

Probability distribution: Normal

Link function: Identity

a. Target: ZAcceptability

Fixed Coefficients

Model Term	Coefficient	Std. Error	t	Sig.	95% CI Lower	95% CI Upper
Intercept	-1.546	.1090	-14.191	.000	-1.760	-1.333
Group=3	1.121	.1578	7.104	.000	.812	1.430
Group=2	1.468	.1804	8.136	.000	1.114	1.822
Group=1	0[b]	.	.	.	.	.
NegMarker=1	1.578	.2013	7.839	.000	1.183	1.973
NegMarker=0	0[b]	.	.	.	.	.
Respuesta_imagen=1	.149	.1042	1.429	.153	-.055	.353
Respuesta_imagen=0	0[b]	.	.	.	.	.
[Group=3]*[NegMarker=1]	-2.394	.2398	-9.983	.000	-2.864	-1.924
[Group=3]*[NegMarker=0]	0[b]	.	.	.	.	.
[Group=2]*[NegMarker=1]	-2.988	.2560	-11.673	.000	-3.490	-2.486
[Group=2]*[NegMarker=0]	0[b]	.	.	.	.	.
[Group=1]*[NegMarker=1]	0[b]	.	.	.	.	.
[Group=1]*[NegMarker=0]	0[b]	.	.	.	.	.
[Group=3]*[Respuesta_imagen=1]	.254	.1510	1.681	.093	-.042	.550
[Group=3]*[Respuesta_imagen=0]	0[b]	.	.	.	.	.
[Group=2]*[Respuesta_imagen=1]	-.012	.1734	-.067	.946	-.352	.328
[Group=2]*[Respuesta_imagen=0]	0[b]	.	.	.	.	.
[Group=1]*[Respuesta_imagen=1]	0[b]	.	.	.	.	.

[Group=1]*[Respuesta_imagen=0]	0[b]	.	.	.	.	.
[NegMarker=1]*[Respuesta_imagen=1]	-.136	.1989	-.683	.495	-.526	.254
[NegMarker=1]*[Respuesta_imagen=0]	0[b]	.	.	.	.	.
[NegMarker=0]*[Respuesta_imagen=1]	0[b]	.	.	.	.	.
[NegMarker=0]*[Respuesta_imagen=0]	0[b]	.	.	.	.	.
[Group=3]*[NegMarker=1]*[Respuesta_imagen=1]	-.235	.2402	-.980	.327	-.706	.235
[Group=3]*[NegMarker=1]*[Respuesta_imagen=0]	0[b]	.	.	.	.	.
[Group=3]*[NegMarker=0]*[Respuesta_imagen=1]	0[b]	.	.	.	.	.
[Group=3]*[NegMarker=0]*[Respuesta_imagen=0]	0[b]	.	.	.	.	.
[Group=2]*[NegMarker=1]*[Respuesta_imagen=1]	.027	.2534	.106	.916	-.470	.524
[Group=2]*[NegMarker=1]*[Respuesta_imagen=0]	0[b]	.	.	.	.	.
[Group=2]*[NegMarker=0]*[Respuesta_imagen=1]	0[b]	.	.	.	.	.
[Group=2]*[NegMarker=0]*[Respuesta_imagen=0]	0[b]	.	.	.	.	.
[Group=1]*[NegMarker=1]*[Respuesta_imagen=1]	0[b]	.	.	.	.	.
[Group=1]*[NegMarker=1]*[Respuesta_imagen=0]	0[b]	.	.	.	.	.
[Group=1]*[NegMarker=0]*[Respuesta_imagen=1]	0[b]	.	.	.	.	.
[Group=1]*[NegMarker=0]*[Respuesta_imagen=0]	0[b]	.	.	.	.	.

Probability distribution: Normal

Link function: Identity

a. Target: ZAcceptability

b. This coefficient is set to zero because it is redundant.

Pairwise Contrasts

Group Pairwise Contrasts	Contrast Estimate	Std. Error	t	df	Adj. Sig.	95% CI Lower	95% CI Upper
Basque - BC-Spa	.025	.087	.292	4778	1.000	-.182	.233
Basque - Cast-Spa	.008	.085	.095	4778	1.000	-.162	.178
BC-Spa - Cast-Spa	-.017	.079	-.218	4778	1.000	-.178	.144

The sequential Bonferroni adjusted significance level is .05.

Confidence interval bounds are approximate.

Pairwise Contrasts

Neg Marker Pairwise Contrasts	Contrast Estimate	Std. Error	t	df	Adj. Sig.	95% CI Lower	95% CI Upper
Without 'ez'/'no' - With 'ez'/'no'	.319	.061	5.262	4778	1.485E-7	.200	.437

The sequential Bonferroni adjusted significance level is .05.

Confidence interval bounds are approximate.

Pairwise Contrasts

SN Interpretation Pairwise Contrasts	Contrast Estimate	Std. Error	t	df	Adj. Sig.	95% CI Lower	95% CI Upper
Double Negation / Incorrect - Single Negation / Correct	-.127	.048	-2.638	4778	.008	-.221	-.033

The sequential Bonferroni adjusted significance level is .05.

Confidence interval bounds are approximate.

Pairwise Contrasts

Neg Marker	Group Pairwise Contrasts	Contrast Estimate	Std. Error	t	df	Adj. Sig.	95% CI Lower	95% CI Upper
With 'ez'/'no'	Basque - BC-Spa	1.513	.119	12.743	4778	.000	1.246	1.779
	Basque - Cast-Spa	1.264	.120	10.493	4778	.000	.976	1.552
	BC-Spa - Cast-Spa	-.249	.092	-2.704	4778	.007	-.429	-.068
Without 'ez'/'no'	Basque - BC-Spa	-1.462	.114	-12.796	4778	.000	-1.718	-1.206
	Basque - Cast-Spa	-1.248	.106	-11.720	4778	.000	-1.503	-.993
	BC-Spa - Cast-Spa	.214	.116	1.846	4778	.065	-.013	.442

The sequential Bonferroni adjusted significance level is .05.

Confidence interval bounds are approximate.

Pairwise Contrasts

Group	Neg Marker Pairwise Contrasts	Contrast Estimate	Std. Error	t	df	Adj. Sig.	95% CI Lower	95% CI Upper
Cast-Spa	Without 'ez'/'no' - With 'ez'/'no'	1.002	.093	10.744	4778	.000	.819	1.185
BC-Spa	Without 'ez'/'no' - With 'ez'/'no'	1.464	.101	14.436	4778	.000	1.266	1.663
Basque	Without 'ez'/'no' - With 'ez'/'no'	-1.510	.118	-12.751	4778	.000	-1.742	-1.278

The sequential Bonferroni adjusted significance level is .05.

Confidence interval bounds are approximate.

Pairwise Contrasts

SN Interpretation	Group Pairwise Contrasts	Contrast Estimate	Std. Error	t	df	Adj. Sig.	95% CI Lower	95% CI Upper
Single Negation / Correct	Basque - BC-Spa	.024	.072	.338	4778	.857	-.122	.170
	Basque - Cast-Spa	-.060	.076	-.791	4778	.857	-.230	.110
	BC-Spa - Cast-Spa	-.084	.076	-1.107	4778	.806	-.267	.098
Double Negation / Incorrect	Basque - BC-Spa	.026	.134	.195	4778	1.000	-.245	.298
	Basque - Cast-Spa	.076	.127	.600	4778	1.000	-.228	.380
	BC-Spa - Cast-Spa	.050	.110	.457	4778	1.000	-.184	.284

The sequential Bonferroni adjusted significance level is .05.

Confidence interval bounds are approximate.

Pairwise Contrasts

Group	SN Interpretation Pairwise Contrasts	Contrast Estimate	Std. Error	t	df	Adj. Sig.	95% CI Lower	95% CI Upper
Cast-Spa	Double Negation / Incorrect - Single Negation / Correct	-.217	.068	-3.172	4778	.002	-.351	-.083
BC-Spa	Double Negation / Incorrect - Single Negation / Correct	-.083	.078	-1.058	4778	.290	-.236	.071
Basque	Double Negation / Incorrect - Single Negation / Correct	-.081	.100	-.808	4778	.419	-.278	.115

The sequential Bonferroni adjusted significance level is .05.

Confidence interval bounds are approximate.

Pairwise Contrasts

SN Interpretation	Neg Marker Pairwise Contrasts	Contrast Estimate	Std. Error	t	df	Adj. Sig.	95% CI Lower	95% CI Upper
Single Negation / Correct	Without 'ez'/'no' - With 'ez'/'no'	.421	.052	8.049	4778	.000	.319	.524
Double Negation / Incorrect	Without 'ez'/'no' - With 'ez'/'no'	.216	.096	2.256	4778	.024	.028	.404

The sequential Bonferroni adjusted significance level is .05.

Confidence interval bounds are approximate.

Pairwise Contrasts

Neg Marker	SN Interpretation Pairwise Contrasts	Contrast Estimate	Std. Error	t	df	Adj. Sig.	95% CI Lower	95% CI Upper
With 'ez'/'no'	Double Negation / Incorrect - Single Negation / Correct	-.024	.067	-.361	4778	.718	-.156	.108
Without 'ez'/'no'	Double Negation / Incorrect - Single Negation / Correct	-.230	.068	-3.362	4778	.001	-.364	-.096

The sequential Bonferroni adjusted significance level is .05.

Confidence interval bounds are approximate.

Pairwise Contrasts

Neg Marker	SN Interpretation	Group Pairwise Contrasts	Contrast Estimate	Std. Error	t	df	Adj. Sig.	95% CI Lower	95% CI Upper
With 'ez'/'no'	Single Negation / Correct	Basque - BC-Spa	1,505	,097	15,516	4778	,000	1,288	1,722
		Basque - Cast-Spa	1,255	,103	12,132	4778	,000	1,007	1,502
		BC-Spa - Cast-Spa	-,250	,107	-2,330	4778	,020	-,461	-,040
	Double Negation / Incorrect	Basque - BC-Spa	1,520	,190	8,016	4778	,000	1,066	1,974
		Basque - Cast-Spa	1,273	,190	6,704	4778	,000	,847	1,699
		BC-Spa - Cast-Spa	-,247	,106	-2,323	4778	,020	-,455	-,039
Without 'ez'/'no'	Single Negation / Correct	Basque - BC-Spa	-1,456	,093	-15,717	4778	,000	-1,664	-1,248
		Basque - Cast-Spa	-1,375	,096	-14,346	4778	,000	-1,604	-1,145
		BC-Spa - Cast-Spa	,081	,094	,869	4778	,385	-,102	,265
	Double Negation / Incorrect	Basque - BC-Spa	-1,468	,180	-8,136	4778	,000	-1,900	-1,036
		Basque - Cast-Spa	-1,121	,158	-7,104	4778	,000	-1,475	-,767
		BC-Spa - Cast-Spa	,347	,184	1,890	4778	,059	-,013	,707

The sequential Bonferroni adjusted significance level is .05.

Confidence interval bounds are approximate.

Pairwise Contrasts

Group	SN Interpretation	Neg Marker Pairwise Contrasts	Contrast Estimate	Std. Error	t	df	Adj. Sig.	95% CI Lower	95% CI Upper
Cast-Spa	Single Negation / Correct	Without 'ez'/'no' - With 'ez'/'no'	1.187	.097	12.211	4778	.000	.997	1.378
	Double Negation / Incorrect	Without 'ez'/'no' - With 'ez'/'no'	.816	.130	6.260	4778	.000	.561	1.072
BC-Spa	Single Negation / Correct	Without 'ez'/'no' - With 'ez'/'no'	1.519	.089	17.088	4778	.000	1.345	1.693
	Double Negation / Incorrect	Without 'ez'/'no' - With 'ez'/'no'	1.410	.158	8.916	4778	.000	1.100	1.720
Basque	Single Negation / Correct	Without 'ez'/'no' - With 'ez'/'no'	-1.442	.085	-16.869	4778	.000	-1.610	-1.275
	Double Negation / Incorrect	Without 'ez'/'no' - With 'ez'/'no'	-1.578	.201	-7.839	4778	.000	-1.973	-1.183

The sequential Bonferroni adjusted significance level is .05.

Confidence interval bounds are approximate.

Pairwise Contrasts

Group	Neg Marker	SN Interpretation Pairwise Contrasts	Contrast Estimate	Std. Error	t	df	Adj. Sig.	95% CI Lower	95% CI Upper
Cast-Spa	With 'ez'/'no'	Double Negation / Incorrect - Single Negation / Correct	-.032	.081	-.392	4778	.695	-.189	.126
	Without 'ez'/'no'	Double Negation / Incorrect - Single Negation / Correct	-.403	.109	-3.684	4778	.000	-.617	-.188
BC-Spa	With 'ez'/'no'	Double Negation / Incorrect - Single Negation / Correct	-.028	.073	-.386	4778	.700	-.172	.115
	Without 'ez'/'no'	Double Negation / Incorrect - Single Negation / Correct	-.137	.139	-.991	4778	.322	-.409	.134
Basque	With 'ez'/'no'	Double Negation / Incorrect - Single Negation / Correct	-.013	.170	-.077	4778	.939	-.347	.321
	Without 'ez'/'no'	Double Negation / Incorrect - Single Negation / Correct	-.149	.104	-1.429	4778	.153	-.353	.055

The sequential Bonferroni adjusted significance level is .05.

Confidence interval bounds are approximate.
